# High-resolution crystal structure of human asparagine synthetase enables analysis of inhibitor binding and selectivity

**DOI:** 10.1038/s42003-019-0587-z

**Published:** 2019-09-17

**Authors:** Wen Zhu, Ashish Radadiya, Claudine Bisson, Sabine Wenzel, Brian E. Nordin, Francisco Martínez-Márquez, Tsuyoshi Imasaki, Svetlana E. Sedelnikova, Adriana Coricello, Patrick Baumann, Alexandria H. Berry, Tyzoon K. Nomanbhoy, John W. Kozarich, Yi Jin, David W. Rice, Yuichiro Takagi, Nigel G. J. Richards

**Affiliations:** 1 School of Chemistry, Cardiff University, Cardiff, UK; 2 Department of Molecular Biology and Biotechnology, University of Sheffield, Sheffield, UK; 3 Department of Biochemistry and Molecular Biology, Indiana University School of Medicine, Indianapolis, IN USA; 4 ActivX Biosciences, Inc, La Jolla, CA USA; 5 Division of Structural Medicine and Anatomy, Kobe University Graduate School of Medicine, Kobe, Japan; 6 Dipartimento di Scienze della Salute “Magna Græcia” di Catanzaro, Viale Europa, 88100 Catanzaro, Italy; 7 Net4Science academic spinoff, Università “Magna Græcia”, Campus Salvatore Venuta, Viale Europa, 88100 Catanzaro, Italy; 8 Department of Biology, California Institute of Technology, Pasadena, CA USA; 9 Foundation for Applied Molecular Evolution, Alachua, FL USA; 10 Present Address: Department of Chemistry and California Institute for Quantitative Biosciences, University of California, Berkeley, CA USA; 11 Present Address: Vividion Therapeutics, San Diego, CA USA

**Keywords:** Computational models, Medicinal chemistry, Enzymes, X-ray crystallography

## Abstract

Expression of human asparagine synthetase (ASNS) promotes metastatic progression and tumor cell invasiveness in colorectal and breast cancer, presumably by altering cellular levels of L-asparagine. Human ASNS is therefore emerging as a *bona fide* drug target for cancer therapy. Here we show that a slow-onset, tight binding inhibitor, which exhibits nanomolar affinity for human ASNS in vitro, exhibits excellent selectivity at 10 μM concentration in HCT-116 cell lysates with almost no off-target binding. The high-resolution (1.85 Å) crystal structure of human ASNS has enabled us to identify a cluster of negatively charged side chains in the synthetase domain that plays a key role in inhibitor binding. Comparing this structure with those of evolutionarily related AMP-forming enzymes provides insights into intermolecular interactions that give rise to the observed binding selectivity. Our findings demonstrate the feasibility of developing second generation human ASNS inhibitors as lead compounds for the discovery of drugs against metastasis.

## Abstract (web summary)

Wen Zhu et al. report the crystal structure of human asparagine synthetase at a 1.85 Å resolution, enabling computational analysis of inhibitor binding. They also find new insights into the intermolecular interactions contributing to binding specificity of inhibitors.

## Introduction

Asparagine synthetase (ASNS) catalyzes the ATP-dependent biosynthesis of L-asparagine in cells from l-aspartic acid using l-glutamine as a nitrogen source^1^. Several recent findings provide evidence for connections between asparagine biosynthesis and human disease, raising the urgency for in-depth studies of human ASNS. First, asparagine synthetase deficiency (ASD), which is a rare human neurological disorder, has been linked to residue changes at several locations throughout the enzyme^2^. Nothing is known about how these ASD-linked changes in ASNS impact catalytic activity and stability of the enzyme, although children with ASD exhibit microcephaly, epileptic-like seizures, and intellectual disability^3^. Second, silencing the gene encoding ASNS inhibits cell proliferation in a murine sarcoma model^4^ generated by oncogenic forms of *Kras*^5,6^. Third, l-asparagine is important for the growth and maintenance of acute lymphoblastic leukemia^7^, and breast^8^, lung^9^, and castration-resistant prostate^10^ cancers. Finally, altering exogenous l-asparagine levels affects tumor cell invasiveness, and enforced expression of human ASNS promotes metastatic progression in both colorectal^11^ and breast cancer^12^ by an undetermined mechanism. All of these observations strongly suggest that human ASNS is a bona fide drug target and that potent, small-molecule ASNS inhibitors will have significant clinical utility in the prevention of metastasis^11,12^, and perhaps more broadly in cancer chemotherapy^13^. In fact, it has been suggested that drugs that alter the availability of asparagine in the body might be useful to treat sarcomas with mutant forms of *Ras*^4^. Access to highly specific, small-molecule ASNS inhibitors that can penetrate cells will be transformative in establishing the feasibility of targeting ASNS as a new strategy to treat recalcitrant cancers.

**Fig. 1:**
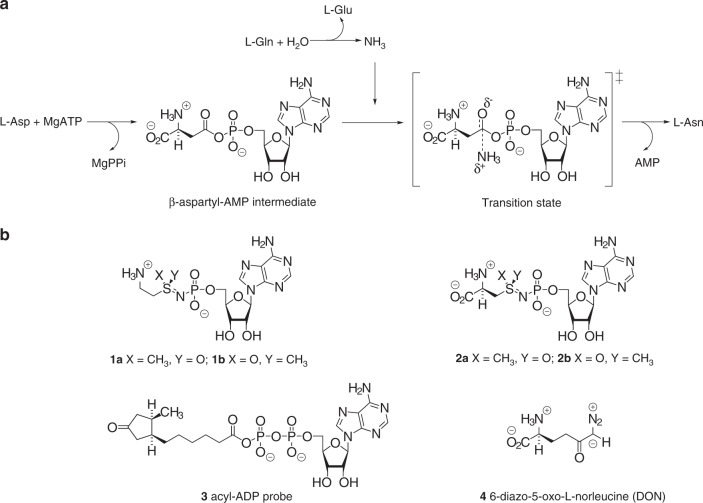
Catalytic mechanism of human ASNS and structures of compounds **1**–**4**. **a** Overview of the chemical transformations catalyzed by ASNS showing the β-aspartyl-AMP intermediate and the transition state for its subsequent reaction with an ammonia molecule, which is released from l-glutamine in a separate glutaminase site. **b** Chemical structures for functionalized methylsulfoximines **1** and **2**, activity-based probe **3** and 6-diazo-5-oxo-l-norleucine **4**

Identifying compounds with nanomolar affinity for human ASNS, however, has proven to be remarkably difficult. The only reported screening studies failed to identify small-molecules with sub-micromolar binding and/or high selectivity for human ASNS^14^, probably because of a lack of mechanistic and structural information about the enzyme. Early work by our group therefore elucidated the kinetic and catalytic mechanisms of the glutamine-dependent asparagine synthetase (AS-B)^15,16^ encoded by the *asnB* gene in *Escherichia coli*^17^. These studies revealed that both the β-aspartyl-AMP intermediate and the transition state for its subsequent reaction with ammonia are tightly bound by the enzyme during catalysis (Fig. 1a)^16^. Although unreactive analogs of the β-aspartyl-AMP intermediate are indeed sub-micromolar ASNS inhibitors^18^, the functionalized methylsulfoximines **1** and **2** (Fig. 1b), which mimic the key transition state for the attack of ammonia on activated esters^19,20^, are slow-onset inhibitors exhibiting nanomolar affinity for the enzyme in kinetic assays^21,22^. Very importantly, ASNS inhibitor **1** (Fig. 1b) negatively impacts the growth of sarcoma cells in a manner similar to that seen when ASNS expression is decreased using siRNA knockdown methods^4^. Moreover, this compound, as a 1:1 mixture of diastereoisomers **1a** and **1b** (Fig. 1b), is cytotoxic against asparaginase-resistant MOLT-4 leukemia cells, albeit only at micromolar concentrations^21^. Unfortunately, although ASNS inhibitor **1** might possess anti-cancer properties, its poor bioavailability curtails its usefulness for studies employing animal models of cancer and metastasis^21^. Nevertheless, this cytotoxic ASNS inhibitor can serve as a starting point for drug discovery. We now report the extent to which ASNS inhibitor **1** participates in off-target binding in HCT-116 cell lysates together with the first high-resolution X-ray crystal structure of human ASNS, to our knowledge. Our studies provide an in-depth understanding of the molecular basis for the specificity of ASNS inhibitor **1** and provide a firm basis for future efforts to generate a second generation of small-molecule ASNS inhibitors with improved bioavailability and reduced chemical complexity.

## Results

### Binding specificity of ASNS inhibitor 1 in HCT-116 lysates

**Fig. 2:**
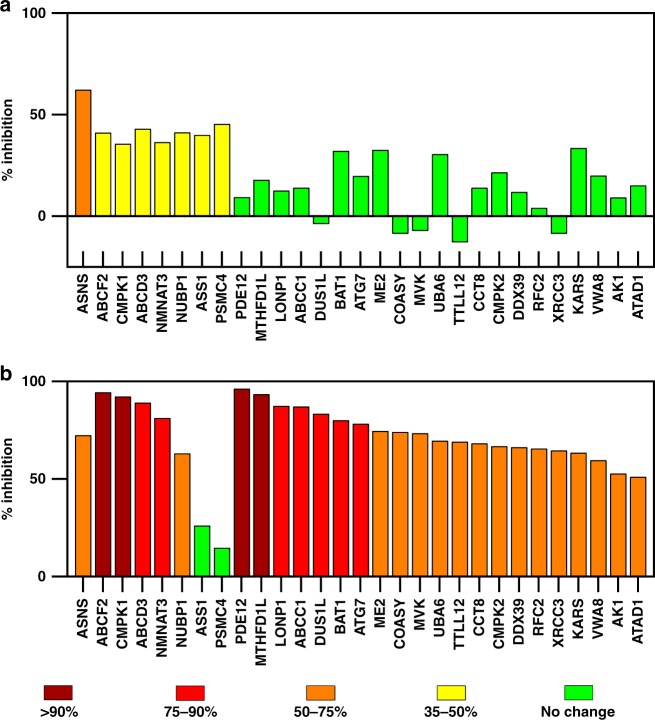
Chemoproteomic Profiling of ASNS Inhibitor 1 Binding to Cellular ATPases. Percentage inhibition of lysine acylation by the reactive probe **3** in the presence of ASNS inhibitor **1** at **a** 10 μM and **b** 100 μM concentration. The inhibition values are color-coded based on suppression of lysine modification, as shown in the legend. Only inhibition levels greater than 50% are considered to be meaningful. Values lower than 35% fall in the range of sample to sample variability (CVs typically 20%). Proteins were initially clustered based on inhibition values for lysates containing 10 μM of inhibitor **1**, and then ranked according to the extent of inhibition measured at 100 μM concentration. Codes for proteins discussed in the text: ASNS–asparagine synthetase; NMNAT3–nicotinate-nucleotide adenylyltransferase; ASS1–argininosuccinate synthetase; KARS–lysyl-tRNA synthetase; CMPK1–UMP-CMP kinase 1; MVK–mevalonate kinase; CMPK2–UMP-CMP kinase 2; AK1–adenylate kinase isoenzyme 1. A complete set of protein codes and chemoproteomic profiling data is provided elsewhere (Supplementary Data 1)

We undertook KiNativ^TM^ chemoproteomic profiling experiments^23^, employing the chemically reactive probe **3** (Fig. 1)^24^, in HCT-116 (ATCC CCL-247) cell lysates to evaluate the affinity of ASNS inhibitor **1** for alternate targets, especially kinases and non-kinase ATPases. This cell line can metastasize in xenograft models and has been used in studies of colon cancer proliferation^25^. In addition, transcriptome profiling indicates that ASNS is expressed in relatively high amounts in HCT-116 cells (Supplementary Fig. 1). Additional support for the cancer relevance of this cell line is provided by recent work showing the importance of asparagine biosynthesis in colorectal cancer cell proliferation and metastasis^11^. Based on the clinically relevant plasma concentration of anticancer drugs^26^, we incubated the probe molecule **3** with HCT-116 cell lysates in the presence (10 μM) or absence of ASNS inhibitor **1**. MS/MS fragmentation and sequence analysis of the tryptic peptides obtained from these reaction mixtures showed that ASNS inhibitor **1** suppressed the ability of probe **3** to acylate the side chain of Lys-466 (located within the ATP-binding site of human ASNS) to an extent of 62% when present in the HCT-116 lysate at 10 μM concentration (Fig. 2). Even though this value is lower than expected, given that ASNS inhibitor **1** is a slow-onset inhibitor^27^ in vitro with a nanomolar K_i_^*^(ref. ^21^), acylation of the active site lysine in ASNS was inhibited to the greatest extent for all ATPases present in the cell lysates. Occupation of the ATP-binding site by ASNS inhibitor **1** will, of course, vary over time as a function of the rate at which the initial EI complex isomerizes to the EI* complex^28^. The complicated environment of the cell lysate makes it difficult to quantify the in vitro isomerization rate constants, but we note that the activity of human ASNS is decreased approximately three-fold after 15 min in the presence of 10 μM ASNS inhibitor **1** under our in vitro assay conditions^21^. In addition, it is also possible that the ability of ASNS inhibitor **1** to bind to the synthetase site of the enzyme is negatively impacted by the ATP concentration in the cell lysates^21^. Importantly, only moderate suppression of lysine acylation by the reactive probe **3** was observed for a small number of off-target enzymes, including nicotinate-nucleotide adenylyltransferase^29^, and argininosuccinate synthetase (ASS1)^30^. There is also a weak interaction of the ASNS inhibitor **1** with the ATP-binding sites of two ABC transporters. On the other hand, 10 μM ASNS inhibitor **1** does not suppress lysine acylation in the ATP-binding sites of phosphopantetheine adenylyltransferase^31^, GMP synthetase^32^, and glutamyl-tRNA synthetase^33^ to any significant extent even though these enzymes also convert ATP to AMP and inorganic pyrophosphate (MgPP_i_) during catalytic turnover (Fig. 2 and Supplementary Data 1). Although the structurally similar compound **2** (Fig. 1b) binds to the *Escherichia coli* ammonia-dependent asparagine synthetase (AS-A)^34^, which is evolutionarily related to bacterial amino-acyl tRNA synthetases^35^, our chemoproteomic profiling studies suggest that ASNS inhibitor **1**, when present in HCT-116 cell lysates at 10 μM concentration, interacts only weakly with lysyl tRNA synthetase and does not bind to seryl or asparaginyl tRNA synthetases (Fig. 2 and Supplementary Data 1). ASNS inhibitor **1** is also bound by UMP-CMP kinase 1 (CMPK1)^36^ and (to a considerably lesser extent) the kinases MVK, CMPK2, and AK1. In efforts to rationalize the interaction of CMPK1 and ASNS inhibitor **1**, and assuming that binding takes place in the ATP-binding site of the kinase, we used manual docking methods to obtain a model of the **1a**/CMPK1 complex based on the X-ray crystal structure of the homologous enzyme in Dictyostelium discoideum^37^ bound to ADP (Supplementary Fig. 2). Although this model suggests that both epimers **1a** and **1b** could possibily interact with the kinase (see Supplementary Information), any in-depth understanding of their binding energetics and intermolecular interactions lies outside the scope of this paper. Given that (i) the chemoproteomic probe **3** can target almost the full complement of human kinases^38^, and (ii) cells generally express about 40% of the human kinome^39^, a high level of selectivity is exhibited by this early-stage AMP-derived inhibitor at 10 μM concentration. This finding therefore supports the idea that a second generation of specific ASNS inhibitors with more drug-like chemical structures^40^ can be developed.

The situation changes somewhat when chemoproteomic assays of HCT-116 lysates are performed in the presence of 100 μM ASNS inhibitor **1** (Fig. 2 and Supplementary Data 1). For example, acylation by the probe **3** is suppressed by over 50% for 29 proteins (Fig. 2), and two- to three-fold increases in inhibition are observed for CMPK1, nicotinamide mononucleotide adenylyltransferase and the ABC transporters. Increased amounts of off-target binding to lysyl tRNA synthetase and other protein kinases in a dose-dependent fashion are also observed. Unexpectedly, suppression of Lys-466 acylation in human ASNS only increases to an extent of 72% when ASNS inhibitor **1** is present at 100 μM concentration (Fig. 2). It is difficult to explain this relatively small increase in the assay although it may reflect changes in binding kinetics due to the increased extent of interaction of the inhibitor **1** with other proteins in the lysate. In addition, because a mixture of epimers **1a** and **1b** is used in the assay, the small change in binding to ASNS may reflect the fact that one of these diastereoisomers has much lower affinity for the enzyme, as discussed below. Finally, the affinity of ASNS inhibitor **1** for both ASS1 and splice isoform 2 of P43686 appears to be substantially decreased under these conditions (Fig. 2). Given that the reasons for this observation were not clear, we evaluated the ability of ASNS inhibitor **1** to bind to human ASS1 using in vitro kinetic measurements. Recombinant ASS1 was obtained by expression in Sf9 cells (Expression Systems, LLC), using the TEQC method for optimizing protein production, and purified by metal affinity chromatography (see Supplementary Information). Incubating the enzyme with substrates in the presence and absence of 10 μM ASNS inhibitor **1** (see Supplementary Information) showed only weak inhibition on the basis of MgPP_i_ formation (Supplementary Fig. 3). ASS1 activity was further decreased but not abolished when the ASNS inhibitor **1** was present at 100 μM concentration. It is therefore possible that weak off-target interactions with ASS1 can take place in the cell.

### Molecular structure of human ASNS

**Fig. 3:**
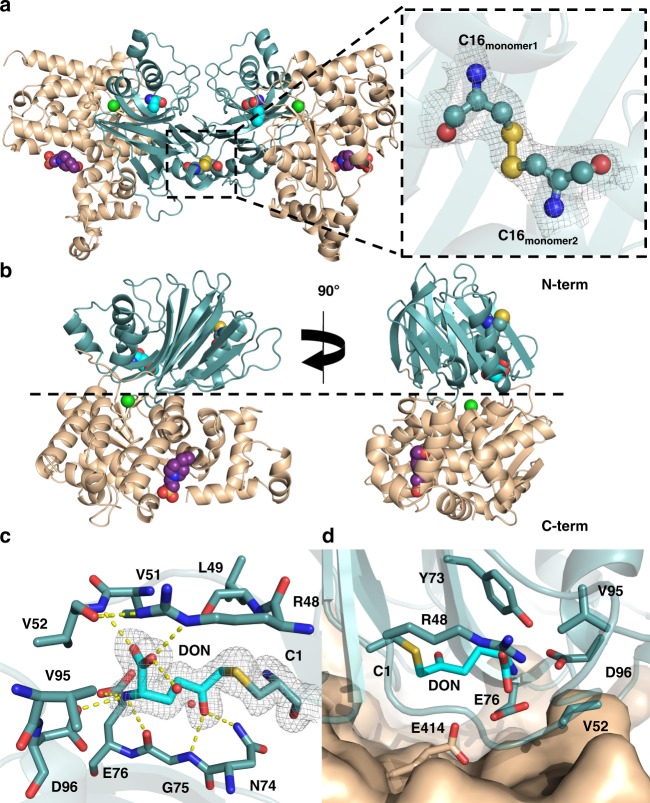
X-ray crystal structure of human ASNS and the glutaminase active site. **a** Cartoon representation of the asymmetric unit of the human ASNS crystal structure and a close-up view of the disulfide bond connecting the N-terminal domains of the ASNS monomers and the electron density surrounding this region (gray mesh, contoured at 0.5 *σ*). The N- and C-terminal domains are colored teal and tan, respectively, and the atoms in the disulfide bond joining the monomers are rendered as spheres. Carbon atoms in the DON moiety and the HEPES molecule present in the synthetase active site are shown as cyan and purple spheres, respectively, and the bound chloride ion is drawn as a green sphere. Atom coloring scheme: C, cyan; N, blue; O, red; S, yellow. **b** Cartoon representation of the human ASNS monomer. The domain and atom coloring scheme is identical to that used in (**a**)**. c** Close-up of DON-modified Cys-1 showing the hydrogen bonding interactions between the DON moiety (cyan) and residues in the glutaminase active site. Electron density for DON, contoured at 0.5 *σ*, is rendered as a mesh. **d** Location of the glutaminase site at the domain/domain interface of human ASNS. Residues are identified using standard one-letter codes and are numbered from the N-terminal residue (Cys-1)

**Fig. 4:**
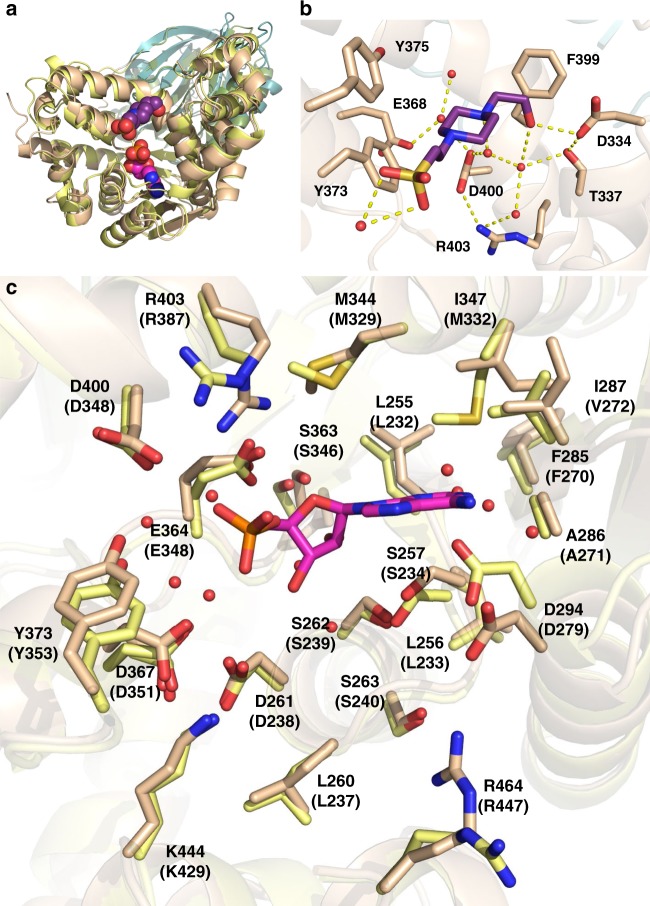
Structural features of the synthetase active site and putative inhibitor binding pocket. **a** Cartoon rendering of DON-modified human ASNS and the C1A AS-B variant (1CT9)^42^ containing a molecule of HEPES (C: purple spheres) and AMP (C: magenta spheres) in the two synthetase active sites, respectively. The N- and C-terminal domains of human ASNS are colored teal and tan, respectively, while the cognate domains in AS-B are colored green and pale yellow, respectively. **b** Close-up view of the network of intermolecular interactions between bound HEPES and residues/water molecules in the synthetase active site of human ASNS. Hydrogen bonds are shown as yellow dashed lines and waters by red spheres. **c** Residue conservation in the synthetase active sites of human ASNS and AS-B with side chain carbons of the two homologs being colored tan and pale yellow, respectively. Carbon atoms in the AMP molecule observed in the AS-B synthetase site are rendered in magenta. Waters are rendered as red spheres. Residues in both structures are identified using one-letter codes and are numbered from the N-terminal residue (Cys-1). AS-B residue numbers are given in parentheses

In order to determine the molecular basis for the binding selectivity of ASNS inhibitor **1** and to provide a firm basis for future, structure-based efforts to identify potent and selective ASNS inhibitors with simplified molecular scaffolds, we determined the high-resolution crystal structure of human ASNS by X-ray crystallography at 1.85 Å resolution (Fig. 3). To date, only the structure of the glutamine-dependent ASNS encoded by the *asnB* gene in *Escherichia coli* has been reported^41^. All efforts to obtain crystals of this and other bacterial ASNS homologs bound to small-molecules other than AMP have failed^42^. Moreover, kinetic studies have shown differences in the ability of ASNS inhibitor **2** (Fig. 1) to bind human ASNS and AS-B^21,43^. For our crystallization experiments, multi-milligram amounts of highly active, recombinant, C-terminally His_10_-tagged, human ASNS were obtained by expression in Sf9 cells^44^ using the TEQC method (see Supplementary Information)^45^. The enzyme was initially purified by metal-affinity chromatography with subsequent removal of the C-terminal His_10_-tag by digestion with the S219P variant of TEV protease^46^. The resulting sample of untagged ASNS was then reacted with DON (6-diazo-5-oxo-l-norleucine)^47^ **4** (Fig. 1a) to modify the reactive thiolate of Cys-1 in the glutaminase active site of ASNS^48^. Mass spectrometric analysis of the as-purified and DON-modified protein showed the DON-modified form of human ASNS to be a homogeneous protein in which other cysteine residues in the protein had not been covalently modified (Supplementary Fig. 4**;** Supplementary Note 1). These mass spectrometric measurements also showed that the N-terminal methionine residue of the recombinant enzyme had been correctly processed. Conditions were then identified that gave a single crystal of the DON-modified enzyme (Supplementary Fig. 5), which diffracted to 1.85 Å resolution (Table 1 and Supplementary Fig. 6). The structure of human ASNS was solved by molecular replacement^49^ using AS-B^41^ as a search model. Two molecules of DON-modified human ASNS were present in the asymmetric unit as a head-to-head dimer in which the two monomers were linked by a disulfide bond that likely forms during crystallization (Fig. 3a). As seen for the bacterial homolog, human ASNS is composed of two domains (Fig. 3b). Residues in the C-terminal (residues 203–560) synthetase domain (41.6% identity) are more conserved than those in the N-terminal (residues 1–202) glutaminase domain (33.9% identity) based on sequence comparisons of human ASNS and its homologs in a number of model organisms (Supplementary Fig. 7). The N-terminal domain of human ASNS possesses the typical sandwich-like α/β/β/α topology present in N-terminal nucleophile (Ntn) amidotransferases^50^, such as GMP synthetase^51^, and glutamine PRPP amidotransferase^52^. A cis-proline (Pro-60) linkage is present in the human enzyme identical to that in the structure of the bacterial homolog AS-B. Electron density for the DON-modified Cys-1 side chain is clearly evident in each monomer (Fig. 3c). A hydrogen bond network, composed of the conserved residues Arg-48, Val-52, Asn-74, Gly-75, Glu-76, and Asp-96, which mediates substrate recognition and thioester stabilization in the hydrolysis reaction that produces ammonia, is also clearly defined^53^. This substrate-binding pocket is located at the interface of the two domains and is within 5 Å of an absolutely conserved glutamate residue (Glu-414) in the C-terminal domain (Fig. 3d). After refinement of the protein and ligands, a single 12 σ peak remained in a pocket at the interface of the N- and C-terminal domains on both chains. The site is surrounded by Tyr-78, Arg-416, Arg-245, and Val-417, and the peak was assigned as a chloride anion (Fig. 3b). The functional importance of this finding remains to be established for human ASNS, but plant asparagine synthetases are known to be activated by chloride^54^. Importantly for structure-based inhibitor discovery, the synthetase site in the C-terminal domain, which is composed of sixteen α-helices and five β-strands, is well resolved (Fig. 3b). We also observed density consistent with a bound HEPES molecule from the crystallization buffer in this domain, which hydrogen bonds to the Asp-334 side chain and water molecules in a network that also involves conserved residues Asp-400 and Arg-403 (Fig. 4a, b). Residues in the synthetase active sites of human ASNS and AS-B are highly conserved (Supplementary Fig. 7) except that Val-272 and Met-333 in the bacterial form of the enzyme are replaced by Ile-287 and Ile-347. Superimposing the human and bacterial structures confirms that the two synthetase active sites are almost identical (Fig. 4c and Supplementary Fig. 8). The Arg-403 side chain, however, adopts different conformations, presumably because AMP is not present in the synthetase site. Interesting structural differences in the intramolecular tunnel connecting the two active sites are also observed for human ASNS and AS-B (Supplementary Fig. 9).

**Table 1:** Crystallographic data collection and refinement statistics

Data collection	
Beamline	Diamond i04
Wavelength (Å)	0.9795
Resolution range (Å)	33.65–1.85 (1.88–1.85)
Space group	P2_1_
Unit cell (a, b, c, α, β, γ)	64.7 Å, 83.52 Å, 110.29 Å, 90°, 90.65^°^, 90^°^
Total reflections	339,772 (16,876)
Unique reflections	100,002 (4932)
Multiplicity	3.4 (3.4)
Completeness (%)	99.8 (99.9)
Mean I/σ (I)	9.7 (1.3)
CC half	0.996 (0.512)
*R*_merge_	0.087 (0.794)
*R*_pim_	0.067 (0.624)

Refinement	
*R*_factor_	0.178
*R*_free_	0.226
RMSD bonds (Å)	0.0119
RMSD angles (°)	1.488
No. of non-H atoms	
Protein	8259
Ligands/metal ions	68
Water molecules	551
Protein residues	1018 (2 chains; A and B)
Average B factors (Å^2^)	
Main chain	31
Side chains	39
Ligands/metal ions	30
Water molecules	31
Ramachandran favored/allowed (%)	97.02/2.78 (Phe399 is an outlier on both chains)
Molprobity score	0.87, 100th percentile (*N* = 12654, 1.85 ± 0.25 Å)

Crystallization conditions: Proplex E9, 0.2 M sodium chloride, 0.1 M Hepes buffer pH 7.5 and 12% PEG 8000. Data collected on 10-07-1016 on MX12788-32 and processed with xia2 -3d pipeline. Data for the highest-resolution shell are given in parentheses. Note that *R*_merge_ = Σ_*hkl*_ Σ_i_ | *I*_i_ – *I*_m_|/Σ_*hkl*_ Σ_i_ *I*_i_ and *R*_pim_ = Σ_*hkl*_ √1/*n* − 1Σ_i=1_ | *I*_i_ − *I*_m_|/Σ_*hkl*_ Σ_i_ *I*_i,_ where *I*_i_ and *I*_m_ are the observed intensity and mean intensity of related reflections, respectively.

### Asparagine synthetase deficiency (ASD) linked variants

**Fig. 5:**
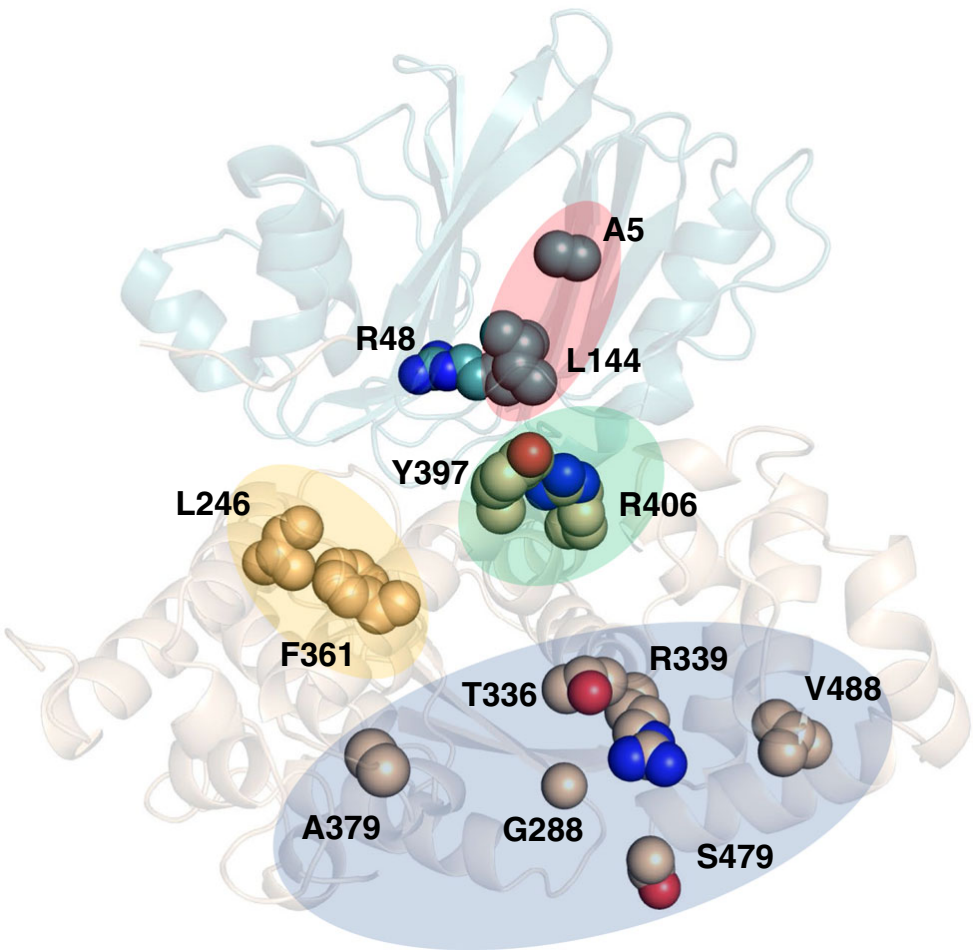
Locations of mutated residues in human ASNS that are associated with asparagine synthetase deficiency. **a** Cartoon representation of the X-ray crystal structure of human ASNS showing the locations of the 15 mutational sites (Ala-5, Arg-48, Leu-144, Leu-246, Gly-288, Thr-336, Arg-339, Phe-361, Ala-379, Tyr-397, Arg-406, Ser-479, Val-488, Trp-540, and Arg-549) that have been identified in patients with asparagine synthetase deficiency. The side chains of mutated residues are rendered as spheres. Colored regions indicate sites that can be classified as Group I (red), Group II (yellow), Group III (green), and Group IV (blue) (Supplementary Table 1)

Having the crystal structure of human ASNS in hand offers an opportunity to map the locations of mutations in 15 residues that have been identified in patients with asparagine synthetase deficiency (ASD) (Fig. 5)^2^. Three of these mutational locations are in the N- terminal domain although, of these, only the Arg-48 side chain is positioned such that it could interact directly with the l-glutamine substrate in the glutaminase active site. Substituting other amino acids in this position might therefore impact the steps leading to ammonia formation. The remaining sites, which are mainly located in the C-terminal domain, can be clustered into four groups (Supplementary Table 1). None of these residues, however, seem to be positioned adjacent to l-aspartate or ATP in the synthetase active site. How these mutations exert their biological effects therefore remains unclear, although these might include altered catalytic activity due to changes in dynamical properties^55^, increased turnover or thermostability of the ASNS variant, or the interaction of ASNS with other proteins within the cell. Based on the ASNS structure we generated the T336I and F361V ASNS variants and assayed their glutamine-dependent activities relative to that of the WT enzyme by measuring MgPP_i_ production (see Supplementary Information). The mutation at Thr-336, a Group IV residue (Supplementary Table 1), which is highly conserved across all the kingdoms (Supplementary Fig. 7), was chosen because it is located at the surface of the C-terminal synthetase active site. Similarly, Phe-361, a Group II residue (Supplementary Table 1), was of interest because it is located in the interior of the C-terminal domain at a distance of 7.2 Å from the chloride-binding site. In addition, Phe-361 is conserved in mammalian asparagine synthetases but is replaced by leucine in the glutamine-dependent plant and bacterial ASNS homologs (Supplementary Fig. 7). Although glutamine-dependent synthetase activity is almost abolished in the T336I ASNS variant, replacing Phe-361 by valine gives an ASNS variant showing a two-fold increase in activity relative to WT enzyme (Supplementary Fig. 10). It is possible that shrinking the Phe-366 side chain may impact structural packing within the C-terminal domain of the enzyme or binding of the chloride anion.

These findings support the idea that some of the ASD-linked changes might perturb asparagine-related metabolism. The apparent involvement of ASNS in neurological development implies that clinically useful ASNS inhibitors against metastasis must not cross the blood-brain barrier or be restricted to use in adults. Conversely, ASNS inhibitors capable of crossing the blood-brain barrier might have clinical utility in treating ASD-linked neurological disorders arising from ASNS variants with enhanced catalytic activity.

### Computational studies of inhibitor binding to human ASNS

**Fig. 6:**
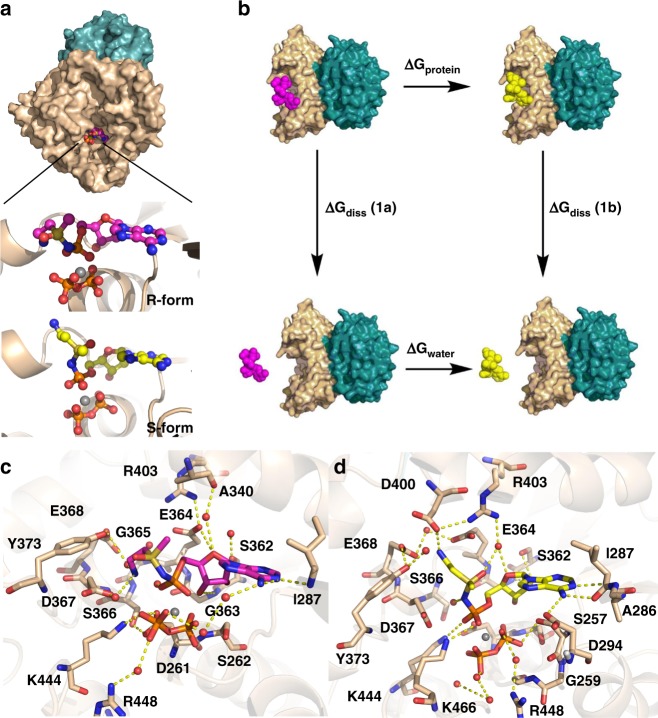
Computational models of the functionalized methylsulfoximines **1a** and **1b** bound within the synthetase active site of the human ASNS/MgPP_i_ complex. **a** Surface representation of human ASNS showing the location of the putative inhibitor binding pocket within the synthetase active site. The N- and C-terminal domains of the enzyme are colored in teal and tan, respectively. Close-up views show the positions of the functionalized methylsulfoximines **1a** (magenta) and **1b** (yellow) relative to the bound inorganic pyrophosphate (orange) and Mg^2+^ (gray) ions in each model complex. **b** Thermodynamic cycle used to estimate the difference in binding free energy (Δ*G*_diss_(**1a**)–Δ*G*_diss_(**1b**)) of the diastereoisomers **1a** and **1b** computed from Δ*G*_protein_–Δ*G*_water_ values obtained by free energy perturbation calculations. Both Δ*G*_diss_(**1a**) and Δ*G*_diss_(**1b**) have positive values since they describe dissociation of each ASNS/ligand complex. **c** Close-up of **1a** showing the non-covalent interactions with synthetase active site residues and water molecules in the computational model. **d** Close-up of **1b** showing the non-covalent interactions with synthetase active site residues and water molecules in the computational model

Kinetic measurements show that **1** is competitive with respect to ATP (the first substrate to bind in the pathway leading to asparagine formation) and can bind to the DON-modified form of human ASNS^21^. Both of these observations are consistent with the idea that ASNS inhibitor **1** binds within the synthetase active site of the enzyme (Fig. 4). Extensive crystallization trials, however, failed to yield crystals of the inhibitor bound to either DON-modified or WT human ASNS. Functionalized sulfoximines are stable to hydrolysis^56^, however, and the ^1^H NMR spectrum of **1** is unchanged over a period of 19 days in the crystallization buffer. Given that epimers **1a** and **1b** cannot be separated by column chromatography, we carried out computational studies on models of human ASNS bound to either **1a** or **1b** in which Cys-1 was present as the unmodified amino acid (Fig. 6a). For these calculations, missing loops in the human ASNS structure, together with residues 534–546 located in the C-terminal tail, were built using the Modeller^57^ and Chimera^58^ software packages, and the conformational properties of these regions were validated with the Discrete Optimized Protein Energy protocol^59^. As a result, only the last 14 residues (547–560) of the enzyme were absent in this model (see Supplementary Information). We then positioned MgPP_i_ within the ASNS synthetase site above a conserved pyrophosphate-binding loop^60^ in an identical orientation to that seen in the X-ray crystal structure of the evolutionarily related enzyme GMP synthetase^51^. MgPP_i_ was included in the model structure because it is the last product released during turnover^16^ and is therefore present in the enzyme when ammonia reacts with the β–aspartyl-AMP intermediate. In silico docking^61^ was used to position β–aspartyl-AMP (Fig. 1a) and each of the epimers **1a** and **1b** into the MgPP_i_/ASNS complex. The resulting three model complexes were next placed in a box of water molecules and subjected to molecular dynamics (MD) simulations (100 ns) (see Supplementary Information).

An extensive series of non-covalent interactions between β–aspartyl-AMP and the enzyme were observed in the equilibrated β–aspartyl-AMP/MgPP_i_/ASNS complex (Supplementary Fig. 11a). For example, the phosphate moiety of this reactive intermediate forms an electrostatic interaction with the side chain of Lys-466, a conserved residue (Supplementary Fig. 7) that is essential for catalytic activity in AS-B^62^. In addition, the 2′-OH group on the ribose ring hydrogen bonds to the side chain of Ser-362, consistent with the fact that dATP is not a substrate for the enzyme. At the other end of the intermediate, the α-amino group of β–aspartyl-AMP forms a salt bridge with the side chain of Asp-367 and the α-carboxylate interacts with Glu-364 via a bridging water molecule. To our knowledge, neither of these residues has been altered previously by site-directed mutagenesis even though both are conserved within known asparagine synthetases (Supplementary Fig. 7). A similar set of interactions to those seen in the β–aspartyl-AMP/MgPP_i_/ASNS complex were found in MD trajectories of the model **1a**/MgPP_i_/ASNS and **1b**/MgPP_i_/ASNS complexes. Importantly for future inhibitor discovery efforts, the positively charged amino group of both **1a** and **1b** is preferentially bound in a pocket defined by the side chains of Glu-364, Asp-367, and Asp-400 (Fig. 5c). Protein/ligand hydrogen bonds are also formed between **1a** or **1b** and Ser-362, Gly-363, and Ile-287. In addition, bound water molecules are predicted to mediate interactions between the functionalized methylsulfoximines and residues Asp-261, Asp-294, Gly-363, and Gly-364 (Supplementary Fig. 11).

Free energy perturbation (FEP/REST2) calculations^63,64^ (see Supplementary Information) were performed to obtain estimates of the free energy difference between the functionalized methylsulfoximines **1a** and **1b** within the synthetase active site of human ASNS (ΔG_protein_) and in water (Δ*G*_water_). Using these values in a thermodynamic cycle (Fig. 5b)^65^ gives an estimate of −10.2 kJ mol^−1^ for (Δ*G*_diss_ (**1a**) − Δ*G*_diss_(**1b**)) (see Supplementary Information), meaning that diastereoisomer **1b** has at least 60-fold greater affinity for ASNS than **1a** at 25 °C. This difference is consistent with the expected value based on qualitative arguments from kinetic data^21,22^.

### A negatively charged cluster underlies binding selectivity

We sought to test these computational models by examining the effect of site-specific mutations on the ability of ASNS inhibitor **1** to bind to human ASNS. Two sets of site-specific mutations were selected on the basis of the intermolecular interactions observed in the computational models of the **1a**/MgPP_i_/ASNS (Fig. 6c) and 1b/MgPP_i_/ASNS complexes (Fig. 6d). Thus, removing the Glu-364 side chain was anticipated to weaken the binding of diastereoisomer **1b** to the enzyme with little, or no, effect on diastereoisomer **1a**. Assuming that **1b** has greater affinity for the enzyme, as suggested by the FEP calculations, the mixture of epimers **1a** and **1b** was anticipated not to inhibit Glu-364 ASNS variants with nanomolar affinity. Similarly, removing the negatively charged Asp-367 side chain was expected to reduce the affinity of **1a** rather than **1b**, meaning that the epimeric mixture **1a** and **1b** would exhibit the same level of inhibition when incubated with Asp-367 ASNS variants.

We expressed and purified ASNS variants in which Glu-364 was replaced by alanine (E364A) or glutamine (E364Q), and Asp-367 was replaced by alanine (D367A) or asparagine (D367N). Kinetic assays monitoring MgPP_i_ formation^21,22^ show that the E364A, E364Q, and D367A ASNS variants are inactive when incubated at pH 8.0 with l-aspartate, ATP, and ammonium chloride as nitrogen source. Efforts to characterize the affinity of the ASNS inhibitor **1** for the three inactive ASNS variants using isothermal calorimetry have been unsuccessful, perhaps because of the slow off-rate of ASNS inhibitor **1** from the E*I complex^21^. These data, however, reveal the importance of Glu-364 in binding and/or catalysis as predicted on the basis of the computational models. Moreover, the D367N ASNS variant exhibits reduced ammonia-dependent activity relative to that of WT enzyme when incubated at pH 8.0 with l-aspartate, ATP and ammonium chloride (Supplementary Fig. 12). The D367N ASNS variant is also inhibited when incubated with 1 μM ASNS inhibitor **1** although MgPP_i_ production is stimulated at short reaction times under these conditions. In addition, inhibition of the D367N ASNS variant is seen at longer times than those at which the inhibitor abolishes ammonia-dependent activity of the WT enzyme (Supplementary Fig. 12). Nevertheless, slow-onset inhibition is still seen for the D367N ASNS variant after 200 sec, perhaps implying that removing the negative charge decreases the isomerization rate of the initial ASNS/inhibitor complex compared to that of WT enzyme. The altered inhibition kinetics support our prediction that epimer **1b** has higher affinity for the synthetase site because of its interaction with the Glu-364 side chain.

### Structure-based insights into ASNS inhibitor selectivity

The synthetase domain of human ASNS is evolutionarily related to similar AMP-forming domains in a large number of other prokaryotic and eukaryotic enzymes^60^. As part of trying to understand the pattern of binding selectivity observed in the chemoproteomic profiling assays (Fig. 2), we identified 128 AMP-forming domains in other protein structures with significant structural similarity to the synthetase domain (residues 222-533) (http://ekhidna2.biocenter.helsinki.fi/dali/)^66^ (Supplementary Data 2). Only 48 of the 128 structural neighbors are present in the human proteome, however, and crystal structures have been deposited for only 10 of these proteins (Supplementary Data 2). Almost all of these enzymes employ ATP, or the structurally similar co-factors SAM and NAD^+^, as substrates, with uroporphyrinogen-III synthase^67^ being an interesting exception. Importantly, transcriptome expression studies show that 47 of these 48 proteins are likely to be present in HCT-116 cell lysates (Supplementary Fig. 1), and we identified peptides from 10 of these proteins in our chemoproteomic profiling assay (Supplementary Data 1). Mechanistic considerations suggest that ASNS inhibitor **1** might exhibit off-target binding to glutamine-dependent NAD^+^ synthetase^68^, GMP synthetase^32,51^, argininosuccinate synthetase^69^, and FMN adenylyltransferase (NMAT1)^70^. Support for this idea is provided by the fact that these human enzymes have AMP-forming domains with the highest structural similarity to the synthetase domain of ASNS (Supplementary Data 2). Of these enzymes, as discussed above, ASNS inhibitor **1** exhibits off-target binding with ASS1 at 100 μM concentration (Supplementary Fig. 3), but there is no evidence to indicate that such an interaction takes place with GMP synthetase in HCT-116 cell lysates (Supplementary Data 1). Tryptic peptides from the ATP-binding sites of glutamine-dependent NAD^+^ synthetase and FMN adenylyltransferase, however, are not observed in the chemoproteomic profiling assays even though both proteins are expressed based on transcriptome data (Supplementary Fig. 1). As a result, the extent to which ASNS inhibitor **1** interacts with these two proteins is unresolved by these chemoproteomic profiling assays.

**Fig. 7:**
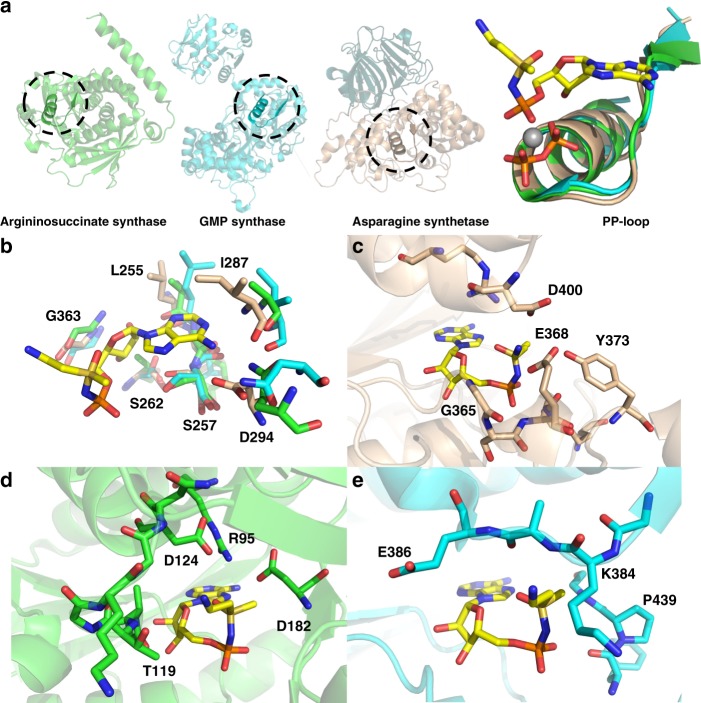
Structural basis for the binding selectivity of ASNS inhibitor **1b**. **a** Alignment of the conserved SGGxD loops (PP-motifs) in argininosuccinate synthetase (2NZ2)^69^ (green), GMP synthetase (2VXO)^32^ (cyan) and the **1b**/MgPP_i_/ASNS computational model (tan). Circles show the location of the PP-motif in the AMP-forming domain of the three enzymes. Carbon atoms in methylsulfoximine **1b** are colored yellow. Color scheme: N, blue; O, red; P, orange; Mg, gray. **b** Superimposition of the homologous AMP-binding sites in argininosuccinate synthetase (C: green), GMP synthetase (C: cyan) and the **1b**/MgPP_i_/ASNS computational model (C: tan) showing the similarity of residues in this region. ASNS residues are labeled using standard one-letter codes and are numbered from the N-terminal residue (Cys-1). **c** Close-up of putative intermolecular interactions between the protonated amino group of **1b** and human ASNS synthetase active site residues (C: tan). ASNS residues are labeled using standard one-letter codes and are numbered from the N-terminal residue (Cys-1). **d** Close-up of argininosuccinate synthetase residues (C: green) surrounding the protonated amino group of **1b** assuming that the ASNS inhibitor binds to the enzyme in a similar pose to that modeled for human ASNS. Argininosuccinate synthetase residues are labeled using standard one-letter codes and are numbered from the X-ray crystal structure^69^. **e** Close-up of GMP synthetase residues (C: cyan) surrounding the protonated amino group of **1b** assuming that the ASNS inhibitor binds to the enzyme in a similar pose to that modeled for human ASNS. GMP synthetase residues are labeled using standard one-letter codes and are numbered from the X-ray crystal structure^32^. The protein orientations in **c**–**e** are aligned to aid structural comparisons

In an effort to place our findings on a structural foundation, we overlaid the conserved PP-loop motifs of our **1b**/MgPP_i_/ASNS model complex and the X-ray crystal structures of human ASS1 and GMP synthetase (Fig. 7a). Even without extensive repositioning of residue side chains, these superimposed structures provide a qualitative picture of active site similarities and differences that might underlie the binding selectivity of ASNS inhibitor **1**. All three enzymes share a common loop motif for binding MgPP_i_ released during formation of the adenylated intermediate (Fig. 7a) and make very similar intermolecular interactions with the AMP moiety of the ASNS inhibitor **1b** (Fig. 7b). Differences in inhibitor binding affinity seem to be associated with a cluster of negatively charged side chains (Fig. 4c) in the ASNS synthetase domain (Glu-364, Asp-367, and Glu-368) that bind the protonated amino group present in ASNS inhibitor **1** on the basis of computational modeling (Fig. 7c). This negatively charged pocket is absent in the active sites of argininosuccinate synthetase (Fig. 7d) and GMP synthetase (Fig. 7e). These data suggest that the residues Glu-364, Asp-367, and Glu-368 define the selectivity of ASNS inhibitor **1** towards human ASNS. If this is the case, second generation ASNS inhibitors must maintain the key electrostatic interactions with this negatively charged pocket if binding specificity is to be realized. In addition, given that the conserved residue Glu-364 is required for catalytic activity, it seems unlikely that resistance mutations could arise at this position in the ASNS synthetase active site.

## Discussion

This work establishes the feasibility of obtaining ASNS inhibitors that exhibit considerable selectivity when present at low, or sub-micromolar concentrations in cells despite the existence of other ATP-utilizing enzymes possessing homologous catalytic domains to ASNS. Access to the X-ray structure of human ASNS, coupled with chemoproteomic profiling, has also allowed us to identify a cluster of negatively charged side chains in the ASNS synthetase domain (Glu-364, Asp-367, and Glu-368) that plays a key role in defining the binding selectivity of ASNS inhibitor **1**. These results will facilitate the discovery of new small-molecule ASNS inhibitors, which can be used (i) to probe the role of l-asparagine production in metastatic progression, and (ii) as agents to control either metastasis and/or tumor growth in animal-based experiments.

## Methods

### Chemical synthesis

Details of the synthetic procedures used to obtain the sulfoximine-based inhibitor **1** have been published elsewhere^21^.

### NMR-based stability studies of ASNS inhibitor 1

A solution of 100 mM sodium HEPES buffer, pH 7.5, containing 200 mM NaCl (540 µL) was added to 10 mM Tris-HCl buffer, pH 8, containing 100 mM NaCl (420 µL) and the mixture was diluted with distilled water (120 µL). An aliquot of this buffer solution (480 µL) was then mixed with 50 mM ASNS inhibitor **1** dissolved in water (20 µL), and the resulting solution was transferred to an NMR tube together with a sealed capillary containing 5.5 mM dimethylmalonate dissolved in D_2_O (60 µL). The final concentration of inhibitor **1** was 1.8 mM. ^1^H NMR spectra were then recorded over a period of 19 days on an Avance-III HD 500 MHz spectrometer. The water signal was suppressed using excitation sculpting with gradients^71^. No changes in the peaks arising from inhibitor **1** were observed during this time even though the sample was stored at room temperature.

### Chemoproteomic profiling

The desthiobiotin-adenosine triphosphate-acylphosphate probe **3** (ATP probe) was synthesized as described previously^24^. An HCT-116 cell pellet was lysed by sonication in lysis buffer (50 mM sodium HEPES, pH 7.5, containing 150 mM NaCl, 0.1% (v/v) Triton X-100 and phosphatase inhibitors [Cocktail II AG Scientific #P-1518]). The samples were then centrifuged in an Eppendorf 5424 R microcentrifuge at 16,200 *xg* for 15 min at 4 °C and the supernatant collected for probe labeling. Five microliters ofthe sulfoximine-based inhibitor **1** was added from 100× stock solutions in dimethyl sulfoxide (DMSO) to 445 μL of lysate in duplicate. DMSO (5 µL) was added in control samples. After 15 min incubation, 50 µL of a 10x aqueous solution of the ATP probe was added to each sample to give a final probe concentration of 5 µM, and samples were incubated with probe for an additional 10 min. Samples were prepared for mass spectrometric (MS) analysis following standard procedures^38^. Briefly, probe-labeled lysates were denatured and reduced in 6 M urea, 10 mM dithiothreitol (DTT) at 65 °C for 15 min), alkylated (40 mM Iodoacetamide, 37 °C, 30 min), and gel filtered (Biorad Econo-Pac® 10 G) into 10 mM ammonium bicarbonate, containing 2 M urea and 5 mM methionine. The desalted protein mixture was digested with trypsin (0.015 mg mL^−1^) for 1 hr at 37 °C, and desthiobiotinylated peptides captured using 12.5 μL high-capacity streptavidin resin (Thermo Scientific). Captured peptides were then washed extensively, and probe-labeled peptides eluted from the streptavidin beads using two 35-μL washes of a 50% (v/v) CH_3_CN/water mixture containing 0.1% (v/v) trifluoroacetic acid (TFA) at room temperature. The resulting samples were then analyzed on Thermo LTQ Velos ion trap mass spectrometers coupled with Agilent 1100 series micro-HPLC systems with autosamplers^72^. For signal extraction and quantitation, up to four ions were typically selected based on their presence, intensity, and correlation to the reference MS/MS spectrum. The resulting chromatographic peaks in each run were then integrated and the integrated peak areas used to determine % inhibition values relative to control samples (Supplementary Data 1).

### Putative interaction of ASNS inhibitor 1 with UMP-CMP kinase 1 (CMPK1)

Given that chemoproteomic profiling showed that ASNS inhibitor **1** binds human CMPK1^36^, which has a completely different fold to the synthetase domain of human ASNS, we built a model of how the ASNS inhibitor **1** might interact with the kinase, making the reasonable assumption that binding takes place within the ATP-binding site. Unfortunately, the X-ray crystal structure of human CMPK1 lacks a bound ligand^73^, and so we superimposed the ASNS inhibitor **1a** on the bound ATP in the active site of the homologous enzyme in *Dictyostelium discoideum*^37^ to obtain an initial model of the **1a**/CMPK1 complex (Supplementary Fig. 2). Preliminary MD simulations of this complex show that if the adenosyl group of **1a** binds to the solvent accessible ATP-binding site then the protonated amino group of the inhibitor prefers to be solvated within the aqueous environment. These calculations also suggest that there are no specific interactions between the sulfoximine moiety and the protein. As a result, we speculate that both epimers **1a** and **1b** could bind to CMPK1. Additional studies, which lie outside the scope of this paper, will be needed to determine exactly how ASNS inhibitor **1** is bound by CMPK1.

### Expression and purification of recombinant human ASNS and the T336I, F361V, E364A, E364Q, D367A, and D367N ASNS variants

The open reading frame (ORF) of human ASNS containing a Tobacco Etch Virus protease (TEV protease)^74^ site (ENLYFQS) followed by a C-terminal 10-histidine tag (His_10_) was codon-optimized, synthesized, and sub-cloned into the *Eco*RV site of a pUC57 vector by GenScript (Piscataway, NJ), to give the pUC57-BamHI-hASNS-TEV-His_10_-HindIII vector (Supplementary Data 3). The *Bam*HI-*Hin*dIII DNA fragment from this vector was then sub-cloned into the same sites in a pFL vector^75^, yielding the pFL-hASNS-TEV-His_10_ vector (pYT1215), which was then used to generate a recombinant baculovirus expressing C-terminally His_10_-tagged human ASNS following published protocols^45^. For large-scale expression, frozen stocks were generated and stored under liquid nitrogen. Sf9 cells were obtained from Expression Systems (Davis, CA), and were maintained in ESF921 medium (Expression Systems) at 27 °C in shaker flasks. The expression of human ASNS in Sf9 cells was optimized using the TEQC method^45^. Briefly, a 1 L culture of Sf9 cells (1.5 × 10^6^ cells mL^−1^) was infected with the baculovirus expressing recombinant human ASNS with MOI = 4.0, the infected cells were then incubated at 27 °C for 96 h before being harvested by centrifugation and frozen in liquid N_2_. The resulting pellet was then stored at −80 °C until lysed in 50 mM Tris-HCl buffer, pH 8.0, containing 500 mM NaCl. After cell lysis and centrifugation, the supernatant was loaded onto a 5 mL GE HisTrap HP column (GE Healthcare) that was pre-equilibrated in 50 mM Tris-HCl buffer, pH 8.0, containing 500 mM NaCl using ÄKTAprime plus FPLC (GE Healthcare) and the C-terminally His_10_-tagged, recombinant human ASNS was eluted from the column with increasing gradient (0–100%) of buffer containing 50 mM Tris-HCl, 500 mM imidazole, 500 mM NaCl, pH 8.0. The fractions containing human ASNS were confirmed by SDS-PAGE and combined. The resulting solution was exchanged to buffer containing 10 mM Tris-HCl buffer, pH 8.0, containing 100 mM NaCl using a PD-10 column (GE Healthcare). Mass spectrometry on the as-purified intact protein confirmed that correctly processed, full-length enzyme had been purified. For crystallography, the C-terminal His_10_-tag was removed using the S219P variant of TEV protease (25:1 molar ratio His_10_-tagged human ASNS:TEV protease) and dialyzed at 4 °C overnight against 25 mM Tris-HCl buffer, pH 8.0, containing 250 mM NaCl and 5 mM DTT. The sample was then flowed over the nickel column again to remove uncleaved His_10_-tagged hASNS and the protease, and the flow-through was exchanged using a PD-10 column (GE Healthcare) into 10 mM Tris-HCl buffer, pH 8.0, containing 100 mM NaCl before the enzyme was concentrated to 6 mg mL^−1^.

In a similar manner, codon-optimized DNAs (Supplementary Data 3) containing mutations encoding the T336I, F361V, E364A, E164Q, D367A, and D367N ASNS variants containing a TEV protease site^74^ followed by a C-terminal 10-histidine tag (His_10_) were synthesized and introduced into pFL-hASNS-TEV-His_10_ vector (pYT1215) by GenScript (Piscataway, NJ) to give vectors pYT1678(T336I), pYT1690(F361V), pYT1668(E364A), pYT1667(E364Q), pYT1670(D367A), and pYT1669(D367N). These transfer vectors were then used to generate baculoviruses that permitted the expression and purification of each human ASNS variant following the same protocol as that outlined above for the untagged, wild-type enzyme.

### Expression and purification of recombinant human argininosuccinate synthetase (ASS1)

The open reading frame (ORF) of human ASS containing an N-terminal His_10_ tag followed by a 3 C protease^76^ site was codon-optimized, synthesized, and sub-cloned into the *EcoRV* site of the pUC57 vector by GenScript (Piscataway, NJ, USA) to give the pUC57-BamHI-hASS-HindIII plasmid (Supplementary Data 3). The *Bam*HI*–Hin*dIII DNA fragment from each vector was then sub-cloned into the same sites in the pKL-10His-3C vector^77^ to give the pKL-10His-3C-hASS (pYT1704) transfer vector. After generation of the baculovirus using this transfer vector, recombinant, N-terminally tagged WT human ASS1 was expressed in Sf9 insect cells using a similar protocol to that described above for WT human ASNS. Cells containing the recombinant enzyme were suspended in 50 mM sodium phosphate buffer, pH 7.5, containing 300 mM NaCl, 10 mM imidazole, 5 mM 2-mercaptoethanol, 0.01% NP-40 and 1x protease inhibitor cocktail (PI). After cell lysis at 4 °C for 30 min, the resulting suspension was centrifuged (35,000 rpm) at 4 °C for 45 min, and the supernatant was incubated with His-Select nickel affinity resin (Sigma) at 4 °C for 1 h. Unbound material was removed by washing the resin with lysis buffer without PI. The recombinant human ASS1 could then be eluted using lysis buffer without PI that contained 500 mM imidazole. Elution fractions were concentrated (Vivaspin, 10000 MW cutoff) to a volume of approximately 1 mL and dialyzed overnight at 4 °C against 50 mM sodium HEPES buffer, pH 7.5, containing 300 mM NaCl, 10% (v/v) glycerol and 2 mM DTT. The sample was then dialyzed at 4 °C against 50 mM sodium HEPES buffer, pH 7.5, containing 300 mM NaCl, 10% (v/v) glycerol and 2 mM TCEP. The resulting sample, which had a final concentration of 0.4 mg mL^−1^, was then stored at −80 °C.

### Kinetic characterization of human ASS1

The activity of recombinant, WT human ASS1 was assayed by measuring the rate of inorganic pyrophosphate (MgPP_i_) production using an enzyme-coupled continuous assay (pyrophosphate reagent P7275, Sigma-Aldrich)^78^. In these experiments, assay mixtures contained 0.5 mM ATP, 0.2 mM l-aspartate, ASS1 (4 μg), pyrophosphate reagent (350 μL), and 2 mM KCl dissolved in 100 mM Tris-HCl buffer, pH 7.5, containing 5 mM MgCl_2_ and ASNS inhibitor **1** (as a 1:1 mixture of diastereoisomers **1a** and **1b**) at 0 μM, 10 μM, or 100 μM concentration (1 mL total volume). Reactions were initiated at 37 °C by the addition of l-citrulline, to a final concentration of 0.2 mM, and NADH consumption was monitored spectrophotometrically at 340 nm over a period of 20 min using a Varian Cary® 50 UV-visible spectrometer (Agilent Technologies) (Supplementary Fig. 3). All kinetic assays were performed in triplicate. A standard curve to convert absorbance units into MgPP_i_ concentration was constructed using known amounts of MgPP_i_ dissolved in 100 mM Tris-HCl buffer, pH 7.5, containing 5 mM MgCl_2_, 0.2 mM l-aspartate, 0.2 mM l-citrulline, and 2 mM KCl. The standard curve was unaffected by the presence of 100 μM ASNS inhibitor **1**.

### Crystallization and structure solution of human ASNS

Prior to crystallization, recombinant human ASNS (140 μL of a 6 mg mL^−1^ solution in 10 mM Tris-HCl buffer, pH 8.0, containing 100 mM NaCl) was incubated with 20 mM 6-diazo-5-oxo-L-norleucine (DON) (20 μL) at room temperature for 45 min to give the DON-modified form of the enzyme (Supplementary Fig. 4)^79^. A solution of 20 mM sodium pyrophosphate (20 μL) was then added to the DON-modified human ASNS to give a stock solution. Automated sitting-drop crystallization trials were carried out on a Hydra II Plus 1 crystallization robot using a 1:1 ratio of protein to reservoir solution. A single crystal grew in conditions containing 0.2 M NaCl, 0.1 M sodium HEPES buffer, pH 7.5, and 12% (w/v) PEG 8000 (ProPlex HT-96, Molecular Dimensions) at 17 °C after a period of 3 weeks (Supplementary Fig. 5). This crystal was harvested after 5 weeks and flash cooled using liquid nitrogen in a cryo-protectant comprising the crystallization conditions, plus an additional 25% (w/v) ethylene glycol. Diffraction data were collected at Diamond Light Source beamline i04 at 100 K, using a wavelength of 0.9795 Å. Data were processed using the 3d module of the Xia2 pipeline^80^ revealing that the crystal belonged to the space group P2_1_ with unit cell dimensions of a = 64.7 Å, b = 83.52 Å, and c = 110.29 Å. Molecular replacement was carried out in PHASER^81^, from within the CCP4i^82^ package, using the glutamine-dependent asparagine synthetase from *Escherichia coli* (PDB:1CT9)^41^ as a search model. Refinement of the initial model was carried out in an iterative manner using REFMAC5 (ref. ^83^) combined with manual rebuilding in Coot^84^. The resulting model, which contained 2 molecules of DON-modified, human ASNS per asymmetric unit, 551 molecules of water, and 2 molecules of HEPES, was refined to 1.85 Å resolution (Supplementary Fig. 6). The MolProbity server^85^ was used to validate the structure, with the Ramachandran plot showing that one residue (Phe-399) is a Ramachandran outlier on both chains. The cognate residue in AS-B is also an outlier, suggesting that an unusual backbone geometry is conserved in this area of the enzyme. All other residues were in favorable or allowed areas of the Ramachandran plot. Coordinates have been deposited in the Protein Data Bank under accession number 6GQ3.

### Kinetic characterization of ASD-linked human ASNS variants

The specific activity of the T336I and F361V ASD-linked ASNS variants was determined using the EnzChek^TM^ Pyrophosphate Assay (Molecular Probes). Briefly, reaction was initiated by addition of 0.1 μM enzyme to a reaction mixture containing 150 mM NaCl, 10 mM MgCl_2_, 1 mM DTT, 10 mM l-glutamine, 10 mM l-aspartate, 5 mM ATP, 0.2 mM MESG, 1 U mL^−1^ purine nucleoside phosphorylase, and 0.03 U mL^−1^ inorganic pyrophosphatase at 37 °C in 100 mM EPPS buffer, pH 8.0. The activity was monitored by measuring the absorption change at 360 nm using a Varian Cary® 50 UV-visible spectrometer (Agilent Technologies). A standard curve was determined using standard MgPP_i_ solution diluted in a buffer solution of 100 mM EPPS, pH 8.0, containing 150 mM NaCl, 10 mM MgCl_2_, 1 mM DTT, 10 mM l-glutamine, 10 mM l-aspartate, 5 mM ATP, 0.2 mM MESG, 1 U mL^−1^ purine nucleoside phosphorylase, and 0.03 U mL^−1^ inorganic pyrophosphatase at 37 °C. The percentage activity of each variant was normalized against that of WT human ASNS (Supplementary Fig. 10).

### Molecular dynamics simulations of human ASNS, its complexes with the β-aspartyl-AMP intermediate, and with the functionalized methylsulfoximines 1a and 1b

The X-ray crystallographic coordinates of DON-modified human ASNS were used to build the initial model of the enzyme for simulations of the enzyme/inhibitor complexes. Segments of missing residues and side chains were built using Modeller^57^ and the Chimera GUI^58^. The conformational properties of these regions were validated with the Discrete Optimized Protein Energy (DOPE) protocol^59^. All amino acid residues introduced into the X-ray crystal structure were geometry optimized with all other atoms constrained to their crystallographic coordinates. This model was then energy minimized following standard protocols to remove bad contacts, before being further refined using the AMBER software suite^86^. Thus, the complex was solvated in a truncated octahedral box (98 × 98 × 98 Å) of TIP3P^87^ water molecules together with eight Na^+^ ions. After assignment of ff14SB force field^88^ parameters, the system was energy minimized while constraining the positions of the non-hydrogen protein atoms (500 kcal mol^−1^ Å^−2^). The restraining force constant was then reduced (10 kcal mol^−1^Å^−2^), and the system slowly heated to 300 K and equilibrated over a period of 2.4 ns in the NVE ensemble with periodic boundary conditions. In these simulations, restraints were placed on hydrogen atoms that were not involved in hydrogen bonds using the SHAKE^89^ algorithm. The temperature of the system in these equilibration simulations was controlled using a Langevin thermostat^90^. A final equilibration was carried out in an NPT ensemble (300 K and 1 atm) for 15 ns. All MD simulations employed a non-bonded cutoff of 12 Å.

This structure was then used as the basis for modeling the **1a**/MgPP_i_/ASNS, **1b**/MgPP_i_/ASNS, and β–aspartyl-AMP/MgPP_i_/ASNS complexes by docking the appropriate ligand into the synthetase active site. The initial positions of the ligands were selected using procedures that we have described elsewhere^21^, with inorganic pyrophosphate being positioned in an identical orientation above the SGGxD loop to that observed in the X-ray crystal structure of GMP synthetase (PDB: 1GPM)^51^. Each of these three structures and the free enzyme were then placed within an orthorhombic box of explicit TIP3P water molecules. The box size was chosen so that there was a 10 Å buffer distance in each dimension from any protein atom. These four model systems were energy minimized, until a gradient threshold of 25 kcal mol^−1^ Å^−1^ was attained, and then equilibrated at 300 K using a series of short MD simulations. The resulting models were then subject to 100 ns of MD simulation in the NPT ensemble at a temperature of 300 K and a pressure of 1 atm using the DESMOND^91^ software package with the OPLS-2005 all-atom force field^92^. In these simulations, hydrogen atoms were constrained using the SHAKE algorithm^89^, and long-range electrostatic energies were computed by particle mesh Ewald^93^ with a short-range cutoff of 12 Å for short-range Coulombic interactions. Structures were sampled from the trajectory at 20 ps intervals (5000 frames per simulation) analyzed using standard methods.

### Free energy perturbation (FEP/REST2) estimates of the relative binding affinities of 1a and 1b

FEP/REST2 calculations^63,64,94^ were performed to obtain estimates of the free energy difference between the functionalized methylsulfoximines **1a** and **1b** within the synthetase active site of human ASNS using the algorithms implemented in the DESMOND^38^ software package and describing atoms in **1a** and **1b** with the OPLS-2005 all-atom force field^39^. Thus, the optimized model of the **1a**/MgPP_i_/ASNS complex was placed within an orthorhombic box (12 Å buffer distance in each dimension from any protein atom) of explicit TIP3P water molecules with sufficient Na^+^ ions to neutralize the charge of the system. Changing the configuration about the sulfur atom in the ligand was accomplished using 100 ns MD simulations at each of 12 λ values (1.0, 0.92, 0.83, 0.75, 0.67, 0.58, 0.42, 0.33, 0.25, 0.17, 0.08, 0.0) with the heavy atoms of the ligand (apart from those of the adenosine moiety) being selected for enhanced sampling, as described elsewhere^63,64,94^. These calculations gave an estimate for Δ*G*_protein_ of −10 ± 2.1 kJ mol^−1^ based on the Bennett acceptance ratio^95^. Following a similar protocol, FEP/REST2 calculations were performed to obtain estimates of the free energy difference between the functionalized methylsulfoximines **1a** (*λ* = 0) and **1b** (*λ* = 1) when solvated in a box of explicit TIP3P water molecules. In these latter set of calculations, the initial conformation of **1a** was identical to that present in the model of the **1a**/MgPP_i_/ASNS complex. These calculations gave an estimate for Δ*G*_water_ of −0.20 ± 0.17 kJ mol^−1^. As a result, the difference in binding free energies of the two ligands is estimated to be −10.2 kJ mol^−1^, based on a standard thermodynamic cycle (Fig. 5b). As a result, one can write the following expression:

$$\Delta G_{{\mathrm{diss}}}\left( {1{\mathbf{a}}} \right) - \Delta G_{{\mathrm{diss}}}\left( {1{\mathbf{b}}} \right) = - 2.303RT\log _{10}\left[ {\frac{{K_{\mathrm{d}}\left( {1{\mathbf{b}}} \right)}}{{K_{\mathrm{d}}\left( {1{\mathbf{a}}} \right)}}} \right]$$

When *R* = 8.314 J mol^−1^ K^−1^ and *T* = 298.15 K, this yields a value of $\frac{{K_{\mathrm{d}}\left( {1{\mathbf{b}}} \right)}}{{K_{\mathrm{d}}\left( {1{\mathbf{a}}} \right)}} = 63$.

### Examining predictions of the computational models by site-directed mutagenesis

The activity of WT human ASNS and four ASNS variants (E364A, E364Q, D367A, and D367N) was assayed by measuring the rate of MgPP_i_ production using a continuous assay employing the EnzChek^TM^ Pyrophosphate Assay (Molecular Probes) as described in detail elsewhere^96^. In these experiments, assay mixtures contained 0.5 mM ATP, 5 mM l-aspartate 0.01 U mL^−1^ nucleoside phosphorylase, 3 × 10^−4^ U mL^−1^ inorganic phosphatase, and 100 mM NH_4_Cl dissolved in 100 mM EPPS buffer, pH 8.0, containing 10 mM MgCl_2_ and either 0 μM or 1 μM ASNS inhibitor **1** (as a 1:1 mixture of epimers **1a** and **1b**) (1 mL total volume). Reactions were initiated at 25 °C by the addition of recombinant, WT human ASNS (4 μg), and 2-amino-6-mercapto-7-methylpurine production was then monitored spectrophotometrically at 360 nm over a period of 10 min. All kinetic assays were performed in triplicate. Identical assay conditions were employed in studies of the D367N ASNS variant except that l-aspartate was present at a final concentration of 50 mM and reactions were initiated by the addition of recombinant enzyme (40 μg). A standard curve to convert absorbance units into MgPP_i_ concentration was constructed using known amounts of MgPP_i_ dissolved in 50 mM EPPS buffer, pH 8.0, containing 50 mM L-aspartate, 200 mM NaCl and 2 mM TCEP. The standard curve was unaffected by the presence of up to 10 μM ASNS inhibitor **1**.

### General note

The pH of all buffer solutions used in the following experimental procedures was adjusted by the addition of either aq. HCl or aq. NaOH.

### Statistics and reproducibility

The primary statistical analysis in the chemoproteomic profiling measurements is a determination of the significance of inhibition using the Student *t*-test. All datapoints for which **1** inhibits modification of the ATP-binding site by the reactive probe **3** to an extent that is greater than 35% are considered significant if *p* < 0.04. Control CVs are calculated using the expression [(control std. deviation)/(average control signal)] * 100%. Estimates of the errors in the free energy calculations are based on the Bennett acceptance ratio. Values for percentage activities or steady-state kinetic parameters are calculated (mean ± standard deviation) from triplicate measurements.

### Reporting summary

Further information on research design is available in the Nature Research Reporting Summary linked to this article.

## Supplementary information

Supplementary Information

Description of Additional Supplementary Files

Supplementary Data 1

Supplementary Data 2

Supplementary Data 3

Reporting Summary

## Data Availability

Atomic coordinates and structure factors for recombinant, DON-modified human ASNS have been deposited in the Protein Data Bank with accession number **6GQ3**. Coordinates for the computational models of the **1a**/MgPP_i_/ASNS, **1b**/MgPP_i_/ASNS and β–aspartyl-AMP/MgPP_i_/ASNS complexes, MD simulation trajectories and I/O files for the free energy calculations, and raw data for protein purification and kinetic assays are available from Professor Nigel Richards (RichardsN14@cardiff.ac.uk) on request. Requests for plasmids and other reagents needed to obtain the ASNS variants used in this study should be sent to Professor Yuichiro Takagi (ytakagi@iu.edu). Raw data for the chemoproteomic profiling experiments can be obtained by contacting Dr. Tyzoon Nomanbhoy (tyzoonn@ACTIVX.com).
