# Author Correction: High-resolution crystal structure of human asparagine synthetase enables analysis of inhibitor binding and selectivity

**DOI:** 10.1038/s42003-019-0690-1

**Published:** 2019-11-22

**Authors:** Wen Zhu, Ashish Radadiya, Claudine Bisson, Sabine Wenzel, Brian E. Nordin, Francisco Martínez-Márquez, Tsuyoshi Imasaki, Svetlana E. Sedelnikova, Adriana Coricello, Patrick Baumann, Alexandria H. Berry, Tyzoon K. Nomanbhoy, John W. Kozarich, Yi Jin, David W. Rice, Yuichiro Takagi, Nigel G. J. Richards

**Affiliations:** 1 School of Chemistry, Cardiff University, Cardiff, UK; 2 Department of Molecular Biology and Biotechnology, University of Sheffield, Sheffield, UK; 3 Department of Biochemistry and Molecular Biology, Indiana University School of Medicine, Indianapolis, IN USA; 4 ActivX Biosciences, Inc, La Jolla, CA USA; 5 Division of Structural Medicine and Anatomy, Kobe University Graduate School of Medicine, Kobe, Japan; 6 Dipartimento di Scienze della Salute “Magna Græcia” di Catanzaro, Viale Europa, 88100 Catanzaro, Italy; 7 Net4Science academic spinoff, Università “Magna Græcia”, Campus Salvatore Venuta, Viale Europa, 88100 Catanzaro, Italy; 8 Department of Biology, California Institute of Technology, Pasadena, CA USA; 9 Foundation for Applied Molecular Evolution, Alachua, FL USA; 10 Present Address: Department of Chemistry and California Institute for Quantitative Biosciences, University of California, Berkeley, CA USA; 11 Present Address: Vividion Therapeutics, San Diego, CA USA

**Keywords:** Computational models, Medicinal chemistry, Enzymes, X-ray crystallography

Correction to: *Communications Biology* 10.1038/s42003-019-0587-z, published online 17 September 2019.

The original version of this Article contained errors in the author affiliations. The affiliations of Adriana Coricello with Dipartimento di Scienze della Salute and Net4Science academic spinoff at Università “Magna Græcia” di Catanzaro were inadvertently omitted. This has now been corrected in both the PDF and HTML versions of the article.

The original version of this Article also omitted the following from the Acknowledgements:

This work was supported by Cardiff University (Y.J., A.R., P.B. and N.G.J.R.), the National Institutes of Health [R01 GM111695 to Y.T.], the National Science Foundation [MCB-1157688 to Y.T.], and the UK Biotechnology and Biological Sciences Research Council (BBSRC) (BB/I003703/1 to D.W.R. and BB/P018017/1 to N.G.J.R.). Funding was also provided by the University of Florida Undergraduate Scholar’s Program (A.H.B.) and a Short-Term Scientific Mission grant from COST Action CA15135 (A.C.).

This has now been corrected in both the PDF and HTML versions of the article.

