# Supplementary material for: High-resolution crystal structure of human asparagine synthetase enables analysis of inhibitor binding and selectivity
Source: Commun Biol. 2019 Sep 17;2:345. doi: 10.1038/s42003-019-0587-z (PMC6748925; doi:10.1038/s42003-019-0587-z)
Supplement: Supplementary file 1 — Supplementary Information [file 42003_2019_587_MOESM1_ESM.pdf]

Supplementary Figures

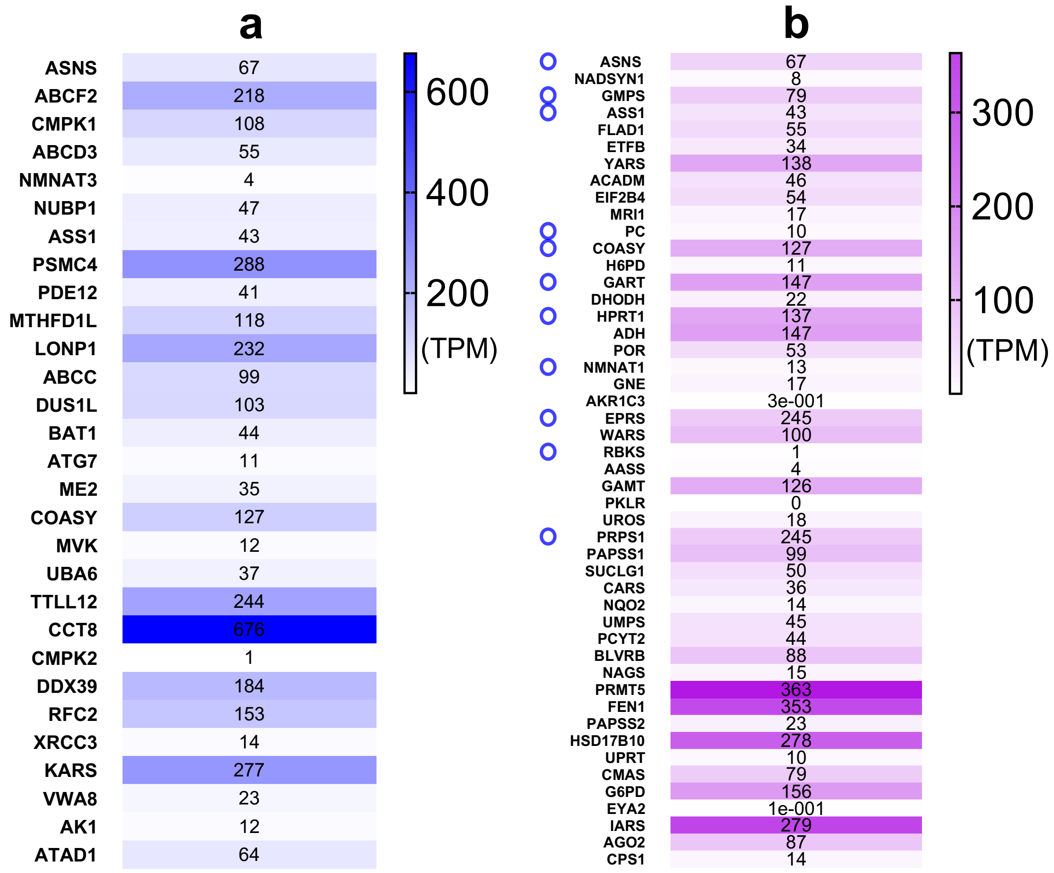

Supplementary Fig 1: RNA-Seq mRNA baseline of HCT-116 cells. (**a**) mRNA expression levels in transcript per million (TPM) for the proteins listed in Supplementary Data 1. (**b**) mRNA expression levels for proteins listed in Supplementary Data 2. In both figures, the color intensity indicates the relative amounts of expression and the number corresponds to the TPM value. Structurally similar proteins to ASNS, which were also identified in the chemoproteomic profiling studies are indicated by blue circles. Data were obtained from the ELIXIR Expression Atlas (https://www.ebi.ac.uk/gxa/home).

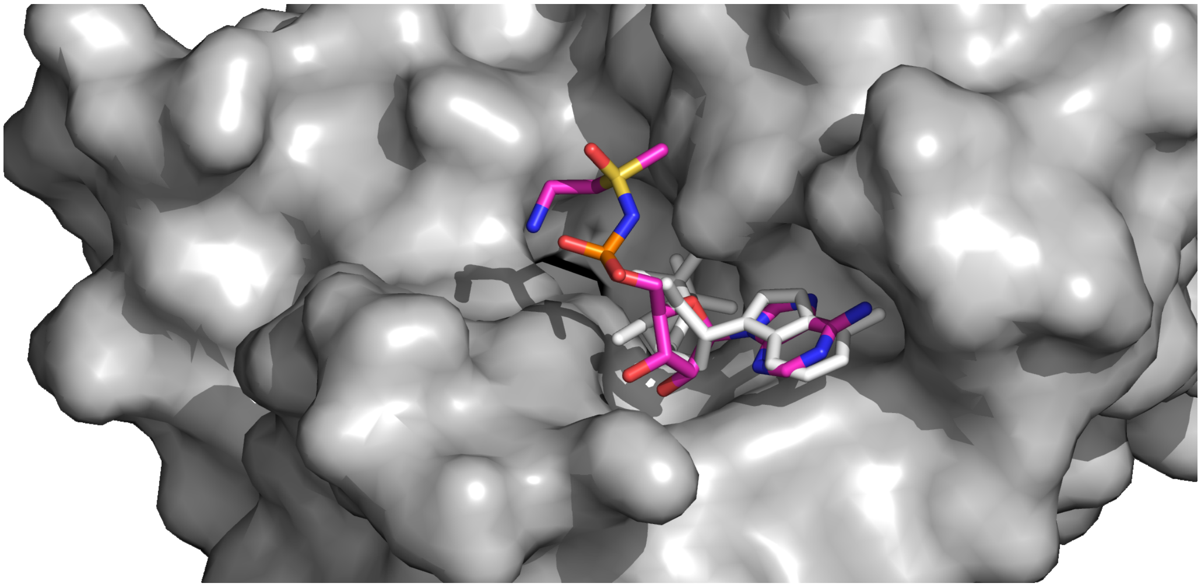

Supplementary Fig 2: Qualitative computational model of ASNS inhibitor 1a bound within the ATP binding pocket of CMPK1. The model structure was generated by superimposing the adenosine moiety of human ASNS inhibitor **1a** (magenta) onto that of bound ADP (white) in the X-ray crystal structure of *Dictyostelium discoideum* UMP-CMP kinase (PDB: 1QF9) (gray surface view)[1], which is a structural homologue of human CMPK1.

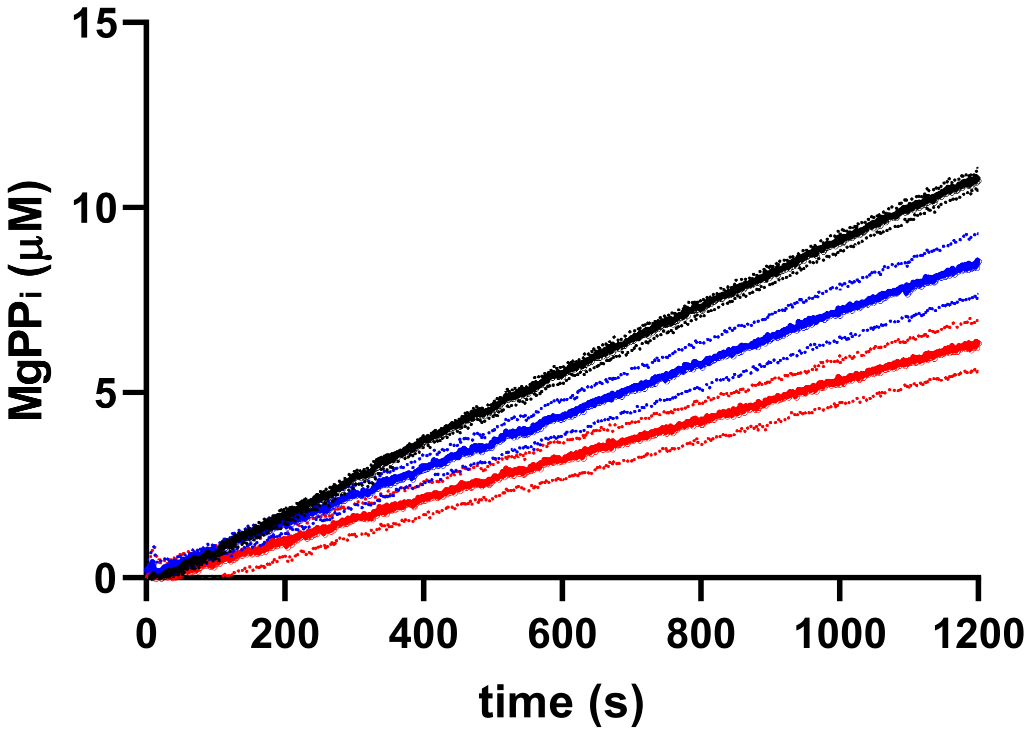

Supplementary Fig. 3: Kinetic characterization of recombinant WT human ASS1 in the presence and absence of ASNS inhibitor 1. Plot of $MgPP_i$ formation as a function of time when recombinant human ASS1 is incubated in the absence (black) and the presence of 10 µM (blue) and 100 µM (red) of human ASNS inhibitor **1**. All measurements were performed in triplicate. The solid lines show the mean value of the three measurements with the standard deviation being rendered as dotted lines.

a

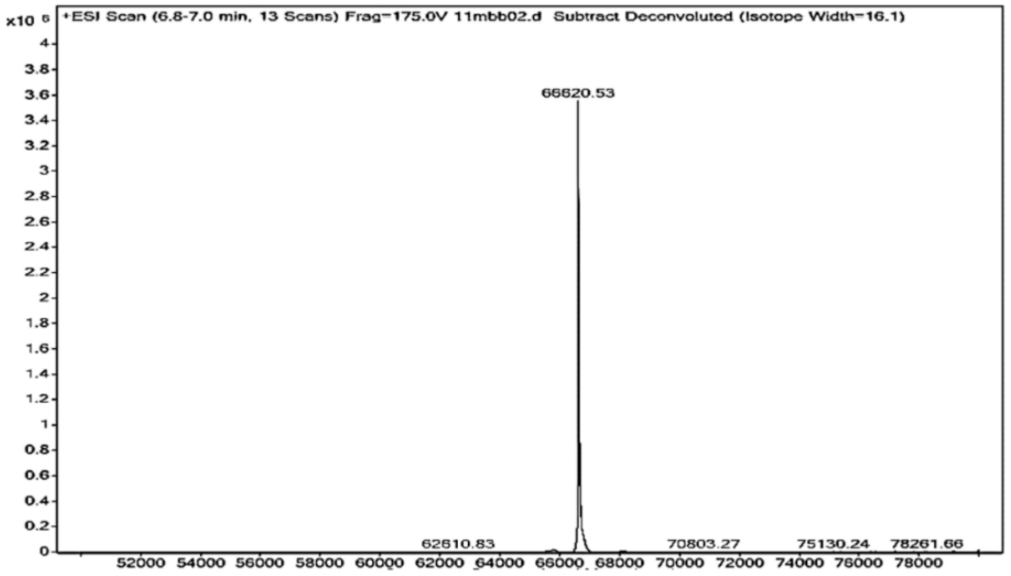

b

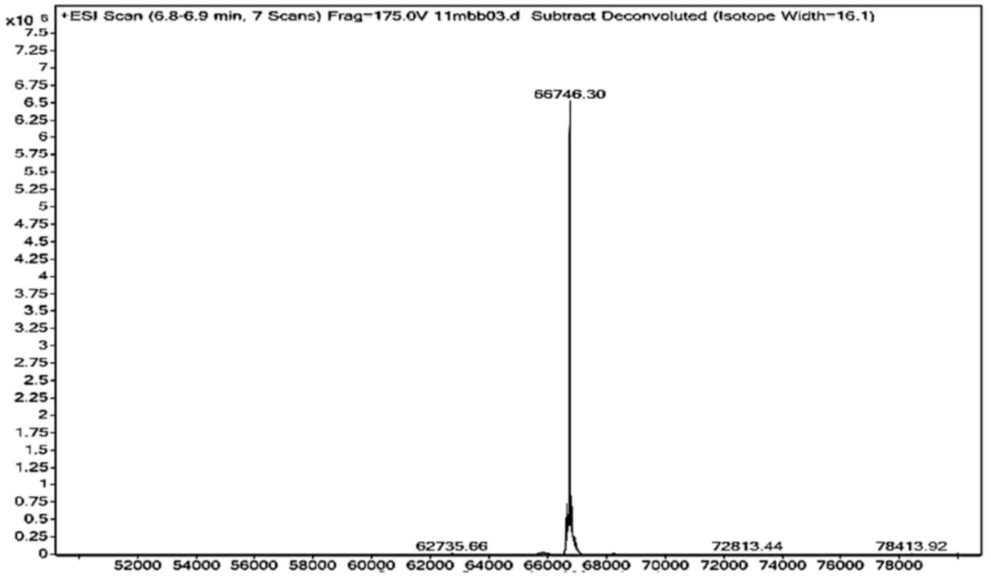

Supplementary Fig. 4: Mass spectrometric analysis of (a) as-purified and (b) DON-modified human ASNS. (**a**) The calculated MW of as-purified His_{10}-tagged human ASNS is 66620.22 Da, which matches well with the observed value of 66620.53 Da. (**b**) The calculated MW for DON-modified His_{10}-tagged human ASNS is 66763.36 Da, which differs from the observed value of 66746.30 Da by 17 mass units; the difference is likely due to the loss of an OH fragment during the ESI-MS measurement. We therefore conclude that only one cysteine is covalently modified by DON in the ASNS monomer.

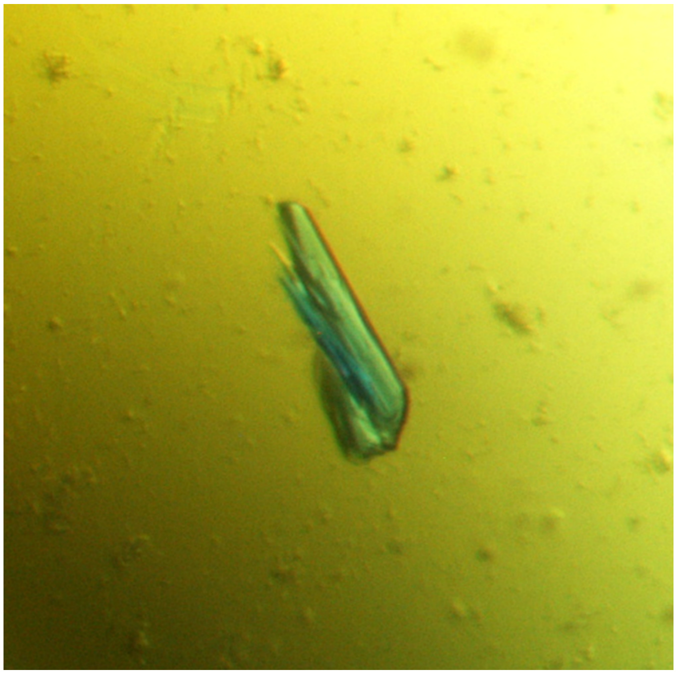

Supplementary Fig. 5: Crystallization conditions for DON-modified human ASNS. After removal of the C-terminal tag by treatment with TEV proteinase, the DON-modified enzyme was prepared by mixing 140 µL of 6 mg mL^{-1} human ASNS and 20 µL of 10 mM DON at room temperature. The reaction mixture was then left for 45 min before the addition of 10 µL of 20 mM potassium phosphate buffer, pH 7.5 (adjusted using aq. NaOH). An aliquot of this solution (0.2 µL) was then mixed with 0.2 µL mother liquor containing 0.2 M sodium chloride dissolved in 0.1 M sodium HEPES, pH 7.5, and 12 % (w/v) PEG 8000. The crystal was harvested after 5 weeks.

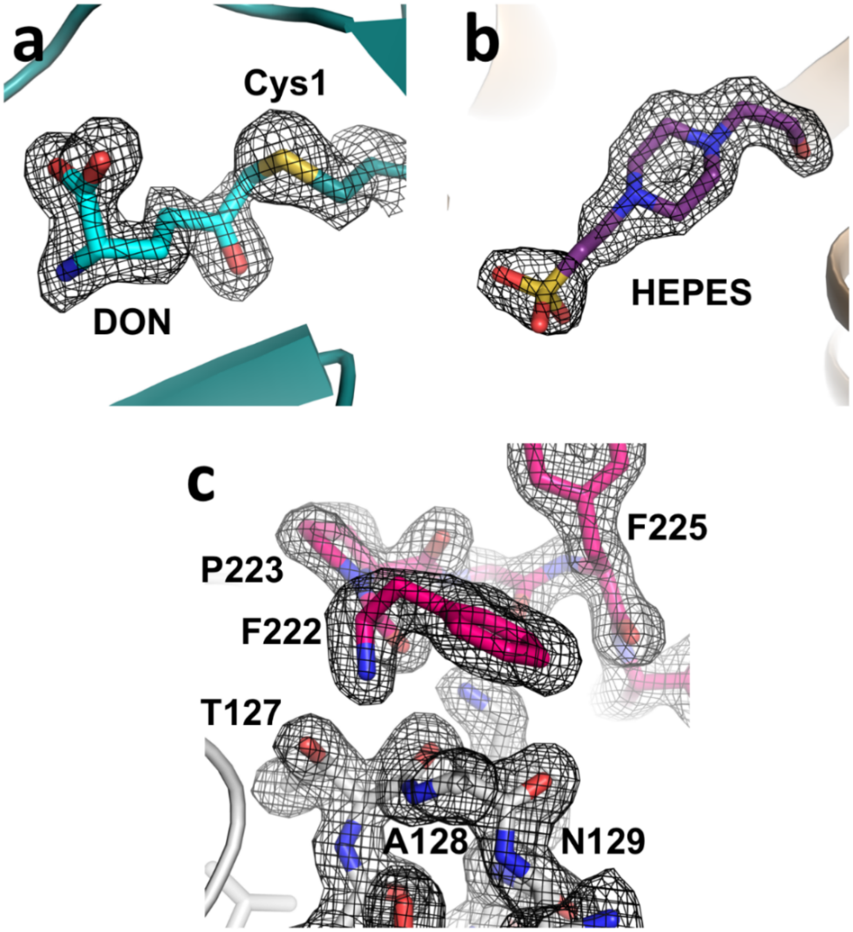

Supplementary Fig. 6: Sample OMIT maps for the X-ray crystal structure of DON-modified human ASNS. (**a**) Omit map for the DON moiety in the glutaminase active site contoured at 1.5σ. (**b**) Omit map for the HEPES molecule bound in the synthetase active site contoured at 1.0σ. (**c**) Omit map for residues at the interface of the two domains of human ASNS contoured at 1.5σ. One monomer is showing in magenta and the adjacent monomer is showing in gray. All three maps were generated with SFCHECK[2].

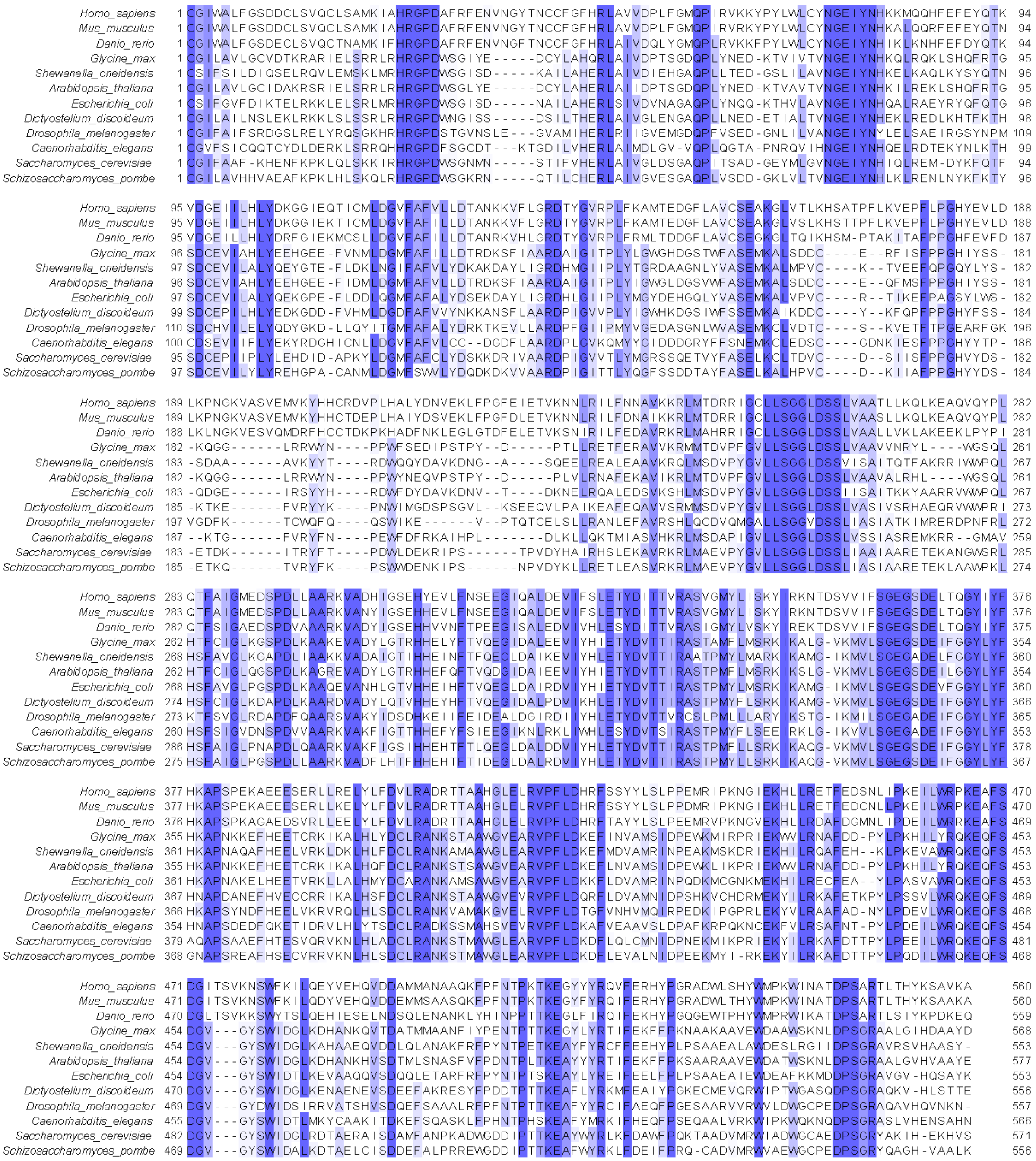

Supplementary Fig. 7: Sequence alignment of asparagine synthetases in selected model organisms[3]. Conserved residues are highlighted in blue. The color intensity is proportional to % conservation.

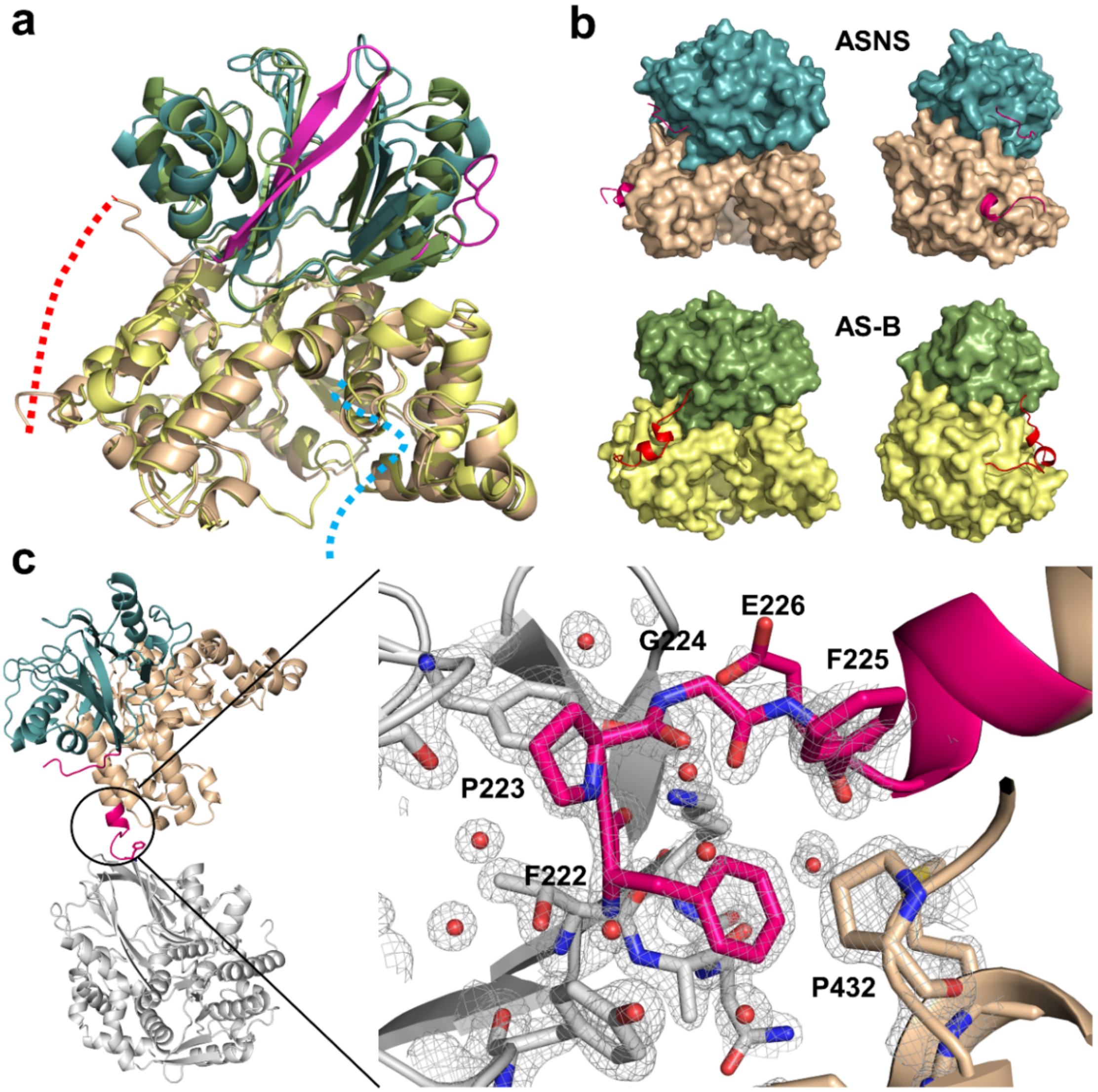

Supplementary Fig. 8: Structural features of human ASNS and comparison with the bacterial homolog AS-B. (**a**) Superimposed X-ray crystal structures of human ASNS (teal/tan) and AS-B (green/pale yellow). The extended loop (residues 166-175) and β hairpin (residues 186-202) regions in human ASNS are colored in magenta. The loop segment (residues 210-221) and C-terminal tail (residues 535-560) that are not observed in the X-ray crystal structure of DON-modified human ASNS are represented by red and cyan dashed lines, respectively. (**b**) Comparison of missing loop segments in ASNS and AS-B. The polypeptides linking the N- and C-terminal domains in human ASNS and AS-B are colored magenta and red, respectively. The proteins are shown in two orientations that differ by a rotation of 90° about the vertical axis. (**c**) Close-up of the interface of two ASNS monomers in the crystal lattice showing the packing of Phe-222. The adjacent monomer is rendered in gray and observed electron density in this region, contoured at 1.5σ is shown as a mesh. Water molecules are rendered as red spheres. The tightly packed C-terminus of the residue Phe-222 suggests that the peptide bond was proteolyzed during the crystallization.

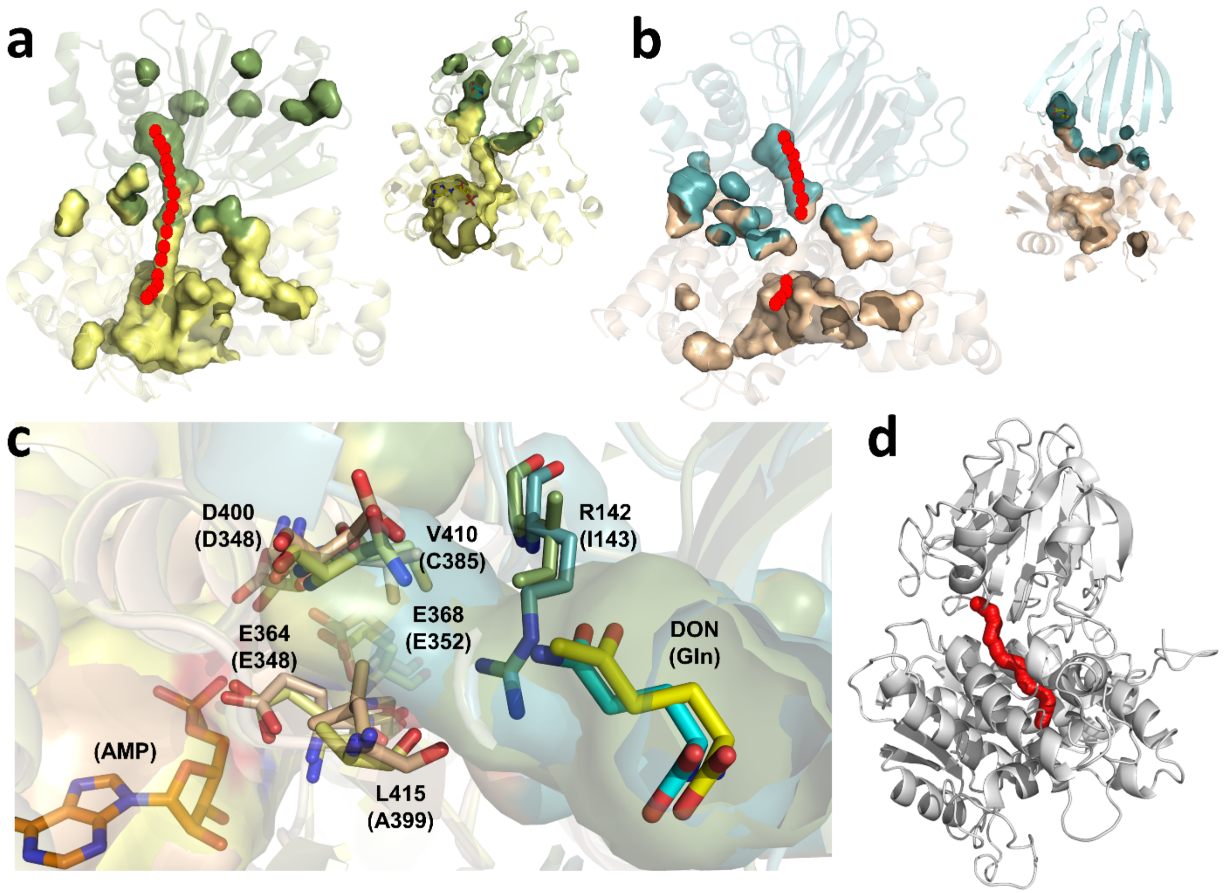

Supplementary Fig. 9: Structural comparison of the ammonia tunnels present in the X-ray crystal structures of DON-modified human ASNS and the C1A variant of *Escherichia coli* AS-B. (**a**) Interior surface of the continuous ammonia tunnel observed in the X-ray crystal structure of AS-B. The N- and C-terminal domains of the bacterial enzyme are colored green and pale yellow, respectively. Red dots indicate the putative path of ammonia as it is travels between the glutaminase and the synthetase active sites. (**b**) Interior surface view of the discontinuous ammonia tunnel observed in the X-ray crystal structure of DON-modified human ASNS. The N- and C-terminal domains of the human enzyme are colored in teal and tan, respectively. Red dots indicate the putative path of ammonia in the two disconnected tunnel regions. (**c**) Superimposition of the X-ray crystal structures of DON-modified human ASNS and *Escherichia coli* C1A AS-B variant showing selected residues defining the putative ammonia tunnels in the two enzymes. The bound L-glutamine and AMP in the AS-B structure are colored in cyan and orange, respectively, and the DON moiety in the human ASNS structure is yellow. (**d**) Location of the putative continuous ammonia tunnel (red dots) linking the glutaminase and synthetase active sites in human ASNS (rendered in gray) based on MD simulations.

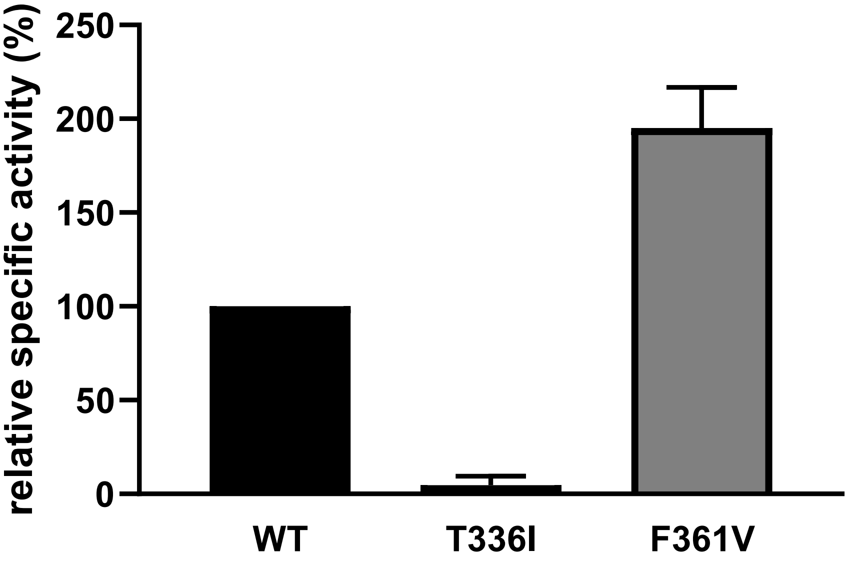

Supplementary Fig. 10: Normalized glutamine-dependent synthetase activities of the T336I and F361V ASNS variants. Catalytic activity was assayed based on the production of $MgPP_i$ using the EnzChek™ Pyrophosphate Assay (Molecular Probes)[4] under the conditions reported above. All measurements were performed in triplicate. Activities of the ASNS variants are expressed relative to that of the WT enzyme (100%).

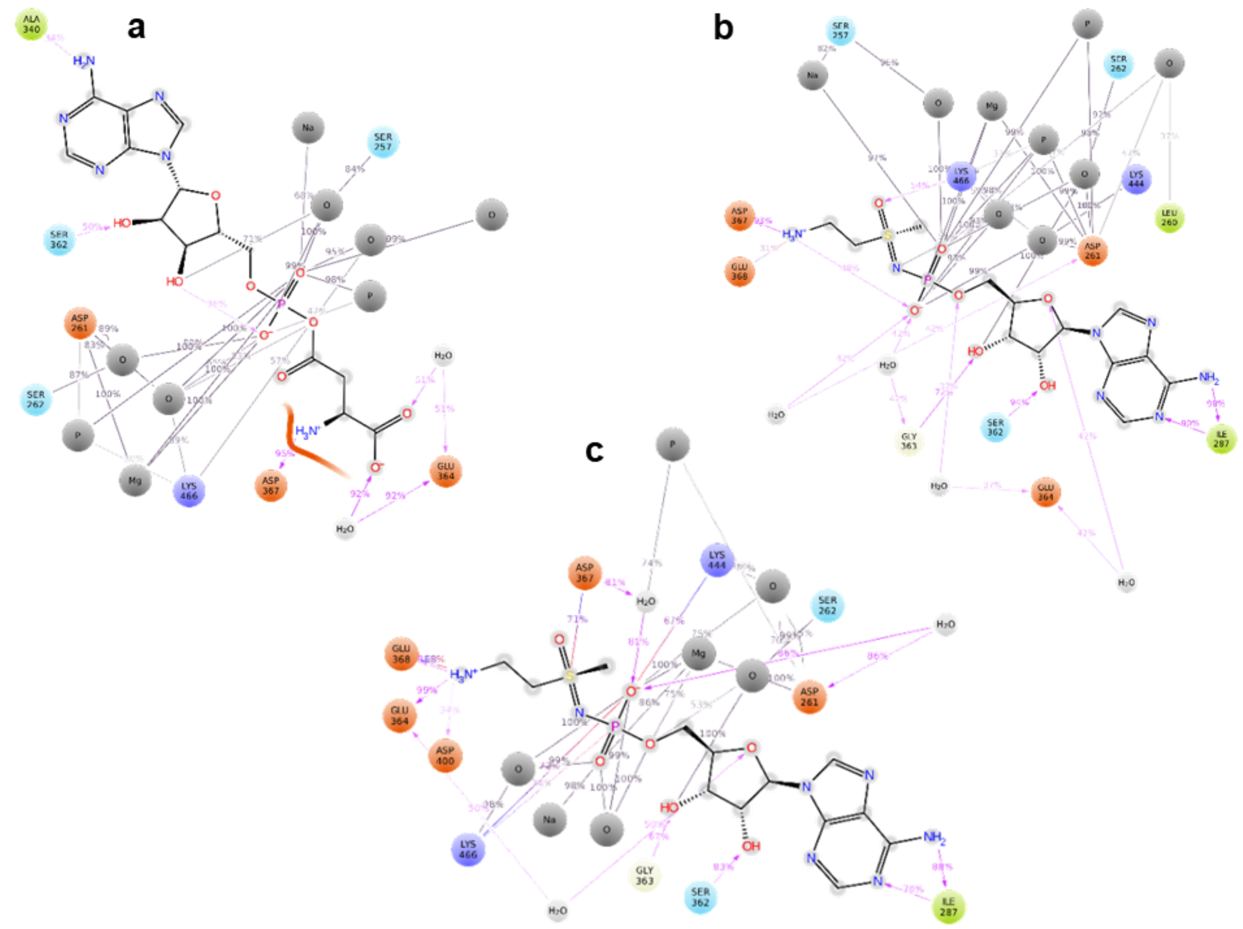

Supplementary Fig. 11: Protein/ligand interactions seen in the MD simulation trajectories. (**a**) Intermolecular interactions observed between protein residues/water molecules in the synthetase active site of human ASNS and β-aspartyl-AMP throughout the MD simulation of the β-aspartyl-AMP/$MgPP_i$/ASNS complex. (**b**) Intermolecular interactions observed between protein residues/water molecules in the synthetase active site of human ASNS and the functionalized methylsulfoximine **1a** throughout the MD simulation of the **1a**/$MgPP_i$/ASNS complex. (**c**) Intermolecular interactions observed between protein residues/water molecules in the synthetase active site of human ASNS and the functionalized methylsulfoximine **1b** throughout the MD simulation of the **1b**/$MgPP_i$/ASNS complex. Percentages show the persistence of a given interaction throughout the sampled structures.

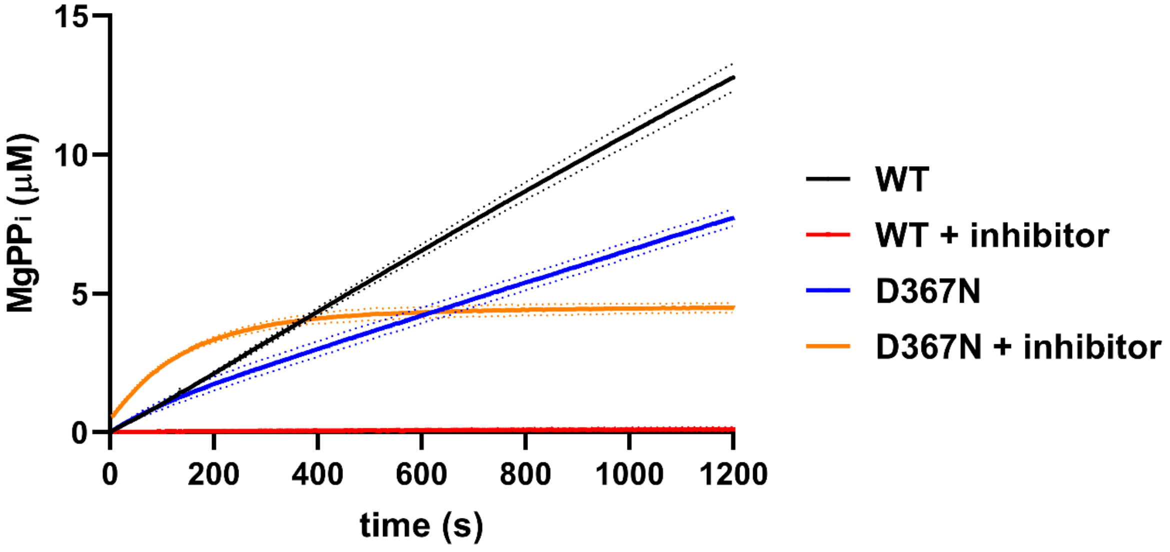

Supplementary Fig. 12: Kinetic characterization of WT ASNS and the D367N ASNS variant in the presence and absence of ASNS inhibitor 1. The time-dependent production of $MgPP_i$ was measured using the EnzChek™ Pyrophosphate Assay (Molecular Probes)[4] under the conditions reported above. The concentrations of WT ASNS and the D367N ASNS variant were 60 nM and 600 nM, respectively, and the ASNS inhibitor **1** was present at 1 µM concentration. All measurements were performed in triplicate. The solid lines show the mean value of the three measurements with the standard deviation being rendered as dotted lines.

Supplementary Table

Supplementary Table 1 Asparagine synthetase deficiency mutation groupings. The disease-related variants are grouped based on the location of the residue that is substituted in protein. Truncated mutations are also observed at Arg-406 and Trp-540.

Group number	Mutations	Notes
N-terminal active site	Arg-48	Conserved residue in the glutaminase active site. Hydrogen bond interaction with L-glutamine.
Group I.	Ala-5<br>Leu-144	Ala-5 and Leu-144 are located in the interface between two halves of the sandwich-like α/β/β/α folding N-terminal domain. Changing this side chain from hydrophobic to hydrophilic could potentially disrupt the packing between the β sheets
Group II.	Phe-361<br>Leu-246	Phe-361 is three amino acids away from a catalytically crucial residue Glu-364. Mutation F361V reduces the side chain size and could alter its hydrophobic interaction with the adjacent residue Leu-246. Alternatively, increasing the side chain volume of Leu-246 in L246W may have the same effect on protein structure and dynamic. Both these two residues also lay closely to the chloride ion in the crystal structure and might also have functional role impact the chloride ion binding or chloride activation that was observed in the plant protein.
Group III.	Tyr-397<br>Arg-406	Tyr-397 and Arg-406 are both located on the interface of domains, which could affect the communication between two domains.
Group IV.	Gly-288<br>Thr-336<br>Arg-339<br>Ser-479<br>Val-488	Gly-288, Thr-336, Arg-339, Ser-479, and Val-488 are all solvent-exposed C-terminal residues. These residues could potentially interact with the unstructured C-terminal tail region and affect enzyme function.
C-terminal tail	Trp-540<br>Arg-549	Absolutely conserved residue in ASNS among different species. Function unknown.

Supplementary Notes

Comparing the structures of DON-modified human ASNS and *Escherichia coli* AS-B. Unlike the bacterial homolog, AS-B, which has shorter and disordered loop, the human enzyme possesses an extended β-strand/turn between residues 186-202 of the N-terminal, glutaminase domain. This hydrogen bond-rich region is not observed in the crystal structure of AS-B, and seems, on the basis of sequence alignment (**Supplementary Fig. 7**), to be a unique feature of asparagine synthetases in primates, rodents and bony fish. Thus, the side chains of Asn-192 and Lys-194 form hydrogen bonds with residues Asn-41, Asp-10 and Asp-151 on the other side of the N-terminal domain, thereby forming a cap bridging the sandwich-like structure of the β-strands (**Supplementary Fig. 8a**). Similarly, residues 166-175 in human ASNS form a longer loop compared to the conjugate segment in AS-B, and this insertion appears to be absent in the homologs found in plants, yeast or bacteria (**Supplementary Fig. 7**). Electron density is missing, however, for residues 210-221 in the polypeptide (203-228) that connects the N- and C-terminal domains of the human enzyme even though the conjugate region in AS-B is well resolved (**Supplementary Fig. 8b**). In addition, the C-terminus of residue Phe-222 in human ASNS is well packed against the N-terminal domain of the adjacent protein monomer in the crystal lattice (**Supplementary Fig. 6c and 8c**). Although it is possible that the lack of electron density for the segment composed of residues 210-221 may be associated with conformational mobility, we note that human ASNS is sensitive to proteolysis. It is therefore entirely possible that this peptide segment (210-221) undergoes proteolysis or hydrolysis during crystallization. Cleavage of this loop may also

be necessary for crystal formation if the presence of the intact loop disrupts the protein-protein contacts needed for lattice stabilization.

The absence of AMP in the synthetase site of human ASNS gives rise to an altered location for the α-helix containing Asp-294 in the two structures. Electron density for residues 468-477 in human ASNS, which also form part of the synthetase site, is poorly defined even though the cognate loop (Lys-449 to Tyr-458) is well defined in the AS-B structure[5]. This suggests that residues on this loop undergo a conformational change when AMP or ATP are bound to the enzyme. Support for this idea is provided by the observation that Lys-449 is important for catalysis and binding of the ASNS inhibitor **1**[(ref. 6)]. As in the case of AS-B, no continuous electron density is observed for the last 26 residues of human ASNS. Once again, the absence of density may reflect the fact that this C-terminal segment is highly disordered. On the other hand, it is also possible that proteolysis takes place during crystallization to remove the C-terminal tail. The likely functional importance of this region, however, is suggested by the fact that residues Trp-536 and Trp-540 are highly conserved, as is the peptide segment DPSxR (x is Gly or Ala), which is present in all asparagine synthetases (**Supplementary Fig. 9**). Searching the sequences of proteins for which structures are available in the Protein Databank revealed that the most conserved region (Tyr-535 to Thr-552) of this disordered segment is also found in the human pancreatic KATP channel (ATP-binding cassette sub-family C member 8, PDB: 6C3P chain E)[7]. We note that this region is also missing from the cryo-EM structure of this channel, which is consistent with the idea that the C-terminal tail of human ASNS is highly flexible. On the other hand, two well resolved crystal structures

(PDB: 2X2S and 4UT1) do contain the DPSxR motif, which is a short α-helix in both proteins[8,9].

As for other glutamine-dependent amidotransferases[10], ammonia formed in the ASNS glutaminase site must be translocated through an intramolecular tunnel to the synthetase active site of the enzyme[11]. In the structure of human ASNS, however, and in contrast to AS-B, the two halves of the ammonia tunnel are disconnected due to the positions of the backbone of Gly-363, Glu-364 and the side chain of Leu-415 (**Supplementary Fig. 9**). The substitution of Ile-143 and Cys-385 (bacterial numbering) in AS-B by Arg-142 and Val-401 (human numbering), respectively, in human ASNS contributes to the tunnel discontinuity seen in our crystal structure. Interestingly, Arg-142 and Val-401 only seem to be present in mammalian asparagine synthetases. We therefore tentatively hypothesize that tunnel opening results from ATP binding given that Glu-364 is located in a mobile loop (residues 361-365). Support for this idea is provided by the X-ray crystal structure of AS-B in which the cognate residue (Glu-348) is hydrogen bonded to the C-3' hydroxyl group of AMP[5]. The functional role of the two site-specific substitutions in facilitating any conformational changes leading to tunnel opening during catalysis remains to be established.

Supplementary References

1. Schlichtling, I. & Reinstein, J. pH influences fluoride coordination number of the AlF_x phosphoryl transfer transition state analog. *Nat. Struct. Mol. Biol.* **6**, 721-723 (1999).
2. Vaguine, A.A., Richelle, J. & Wodak, S. SFCHECK: a unified set of procedures for evaluating the quality of macromolecular structure-factor data and their agreement with atomic models. *Acta Crystallogr. D: Biol. Crystallogr.* **55**, 191-205 (1999).
3. Altschul, S.F., Madden, T.L., Schäffer, A.A., Zhang, J., Zhang, Z., Miller, W. & Lipman, D.J. Gapped BLAST and PSI-BLAST: a new generation of protein database search programs. *Nucl. Acids Res.* **25**, 3389-3402 (1997).
4. Upson, R.H., Haugland, R.P., Malekzadeh, M.N. & Haugland, R.P. A spectrophotometric method to measure enzymatic activity in reactions that generate inorganic pyrophosphate. *Analyt. Biochem.* **243**, 41-45 (1996).
5. Larsen, T.M., Boehlein, S.K., Schuster, S.M., Richards, N.G.J., Thoden, J.B., Holden, H.M. & Rayment, I. Three-dimensional structure of *Escherichia coli* asparagine synthetase B: A short journey from substrate to product. *Biochemistry* **38**, 16146-16157 (1999).
6. Ikeuchi, H., Meyer, M.E., Ding, Y., Hiratake, J. & Richards, N.G.J. A critical electrostatic interaction mediates inhibitor recognition by human asparagine synthetase. *Bioorg. Med. Chem.* **17**, 6641-6650 (2009).
7. Lee, K., Chen, J. & MacKinnon, R. Molecular structure of human KATP in complex with ATP and ADP. *eLife* **6**:e32481 (2017).
8. Schulzenbacher, G., Roig-Zamboni, V., Peumans, W.J., Rougé, P., Van Damme, E.J. & Bourne, Y. Crystal structure of the GalNAc/Gal-specific agglutinin from the

phytopathogenic ascomycete *Sclerotinia sclerotiorum* reveals novel adaptation of a β-trefoil domain. *J. Mol. Biol.* **400**, 715-723 (2010).

9. Gourlay, L.J., Thomas, R.J., Peri, C., Conchillo-Solé, O., Ferrer-Navarro, M., Nithichanon, A., Vila, J., Lertmemongkolchai, G., Titball, R., Colombo, G. & Bolognesi, M. From crystal structure to *in silico* epitope discovery in the *Burkholderia pseudomallei* flagellar hook-associated protein FlgK. *FEBS J.* **282**, 1319-1333 (2015).
10. Huang, X.-Y., Holden, H.M. & Raushel, F.M. Channeling of substrates and intermediates in enzyme-catalyzed reactions. *Annu. Rev. Biochem.* **70**, 149-180 (2001).

11. Li, K.K., Beeson, W.T. & Richards, N.G.J. A convenient gHMQC-based NMR assay for investigating ammonia channeling in glutamine-dependent amidotransferases: Studies of *Escherichia coli* asparagine synthetase B. *Biochemistry* **46**, 4840-4849 (2007).

Data availability

Atomic coordinates and structure factors for recombinant, DON-modified human ASNS have been deposited in the Protein Data Bank with accession number **6GQ3**. Coordinates for the computational models of the **1a**/$MgPP_i$/ASNS, **1b**/$MgPP_i$/ASNS and β–aspartyl-AMP/$MgPP_i$/ASNS complexes, MD simulation trajectories and I/O files for the free energy calculations, and raw data for protein purification and kinetic assays are available from Professor Nigel Richards (RichardsN14@cardiff.ac.uk) on request. Requests for plasmids and other reagents needed to obtain the ASNS variants used in this study should be sent to Professor Yuichiro Takagi (ytakagi@iu.edu). Raw data for the chemoproteomic profiling experiments can be obtained by contacting Dr. Tyzoon Nomanbhoy (tyzoonn@ACTIVX.com).
