# Supplementary material for: High-resolution crystal structure of human asparagine synthetase enables analysis of inhibitor binding and selectivity
Source: Commun Biol. 2019 Sep 17;2:345. doi: 10.1038/s42003-019-0587-z (PMC6748925; doi:10.1038/s42003-019-0587-z)
Supplement: Supplementary file 2 — Description of Additional Supplementary Files [file 42003_2019_587_MOESM2_ESM.docx]

**Description of additional supplementary items**

**Supplementary Data 1**

**Full data set for the chemoproteomic profiling assay showing the % inhibition of cellular ATPases by the ASNS inhibitor 1 at 10 μM and 100 μM concentration.**

% Inhibition values are color-coded to show the strength of the inhibition based on suppression of lysine modification by the reactive probe **3** in the presence of ASNS inhibitor **1**. Inhibition levels higher than 50% are considered meaningful while levels lower than 35% show no binding of the inhibitor **1** to the ATP binding site. All proteins detected in the assay were initially clustered based on the extent of inhibition when ASNS inhibitor **1** was at 10 μM concentration. Within each of the resulting clusters, proteins were ordered according to % inhibition 100 μM ASNS inhibitor **1**. Proteins for which the peptide data sets failed the Student *t*-test (no colored column) are shown at the end of the list. (Provided as a separate Excel spreadsheet)

**Supplementary Data 2**

**Full data set for proteins possessing domains with structural similarity to the ASNS synthetase domain (222-533) as identified by the DALI server.**

Proteins are ordered based on the z value in the DALI search. Proteins for which there are observable tryptic peptide fragments in the chemoproteomic profiling measurements on HCT-116 cell lysates are indicated in the table. Note that crystal structures are not available for the human homologues of several proteins identified in the structure similarity search. In those cases, the reported z-value is for the structure deposited into the PDB. (Provided as a separate Excel spreadsheet)

**Supplementary Data 3**

**Oligonucleotide sequences**

(Provided as a separate Word document)
