# Supplementary material for: High-resolution crystal structure of human asparagine synthetase enables analysis of inhibitor binding and selectivity
Source: Commun Biol. 2019 Sep 17;2:345. doi: 10.1038/s42003-019-0587-z (PMC6748925; doi:10.1038/s42003-019-0587-z)
Supplement: Supplementary file 5 — Supplementary Data 3 [file 42003_2019_587_MOESM5_ESM.docx]

**Oligonucleotide Sequences**

**WT Human ASNS-TEV-His_10_**

ATGTGTGGTATTTGGGCATTGTTCGGCAGCGACGATTGCTTGTCAGTCCAGTGTCTGTCAGCCATGAAGATTGCTCACCGTGGTCCTGACGCATTCCGTTTCGAAAACGTCAACGGATACACTAACTGCTGTTTCGGTTTCCACAGATTGGCTGTGGTCGACCCTCTGTTCGGTATGCAGCCCATCCGCGTGAAGAAATACCCCTACCTGTGGCTCTGCTACAACGGCGAGATCTACAACCACAAGAAAATGCAGCAACATTTCGAATTCGAGTACCAGACTAAGGTGGACGGAGAAATCATTTTGCACCTGTACGATAAGGGTGGCATCGAGCAAACAATTTGTATGCTGGACGGTGTCTTCGCATTCGTTCTGCTCGATACAGCGAACAAGAAAGTGTTCCTGGGTCGTGACACGTACGGCGTCAGGCCTCTCTTCAAGGCCATGACCGAAGATGGCTTCTTGGCTGTCTGCAGTGAGGCCAAGGGACTGGTTACCCTCAAACACTCGGCTACTCCTTTCCTCAAGGTCGAACCTTTCTTGCCCGGTCATTACGAGGTTCTCGACTTGAAGCCCAACGGCAAAGTCGCATCTGTTGAAATGGTGAAGTACCACCATTGCCGCGACGTTCCCTTGCACGCGCTGTACGATAACGTGGAGAAGCTGTTCCCAGGCTTCGAAATCGAGACGGTTAAGAACAACCTCCGTATTTTGTTCAACAACGCTGTGAAGAAAAGGCTGATGACAGACCGCCGTATCGGATGTTTGCTGAGTGGAGGTCTGGATTCCAGCCTCGTCGCTGCCACGCTCTTGAAGCAGCTCAAAGAAGCTCAGGTTCAATACCCATTGCAAACCTTCGCCATCGGTATGGAGGACTCACCGGATCTGCTCGCAGCGCGCAAGGTGGCTGACCACATCGGTTCCGAACATTACGAGGTCCTGTTCAACAGCGAGGAAGGTATCCAGGCTTTGGACGAAGTCATTTTCTCTCTGGAGACATACGATATCACCACTGTGCGTGCCTCTGTCGGAATGTACCTCATCTCAAAGTACATTAGGAAAAACACTGACTCAGTTGTGATCTTCAGTGGCGAAGGATCGGATGAGCTGACACAAGGCTACATTTACTTCCACAAGGCTCCAAGTCCGGAAAAAGCCGAGGAAGAGTCGGAAAGGTTGCTGAGAGAGCTGTACCTCTTCGACGTCTTGAGAGCAGATCGTACAACGGCTGCACACGGATTGGAGCTGAGAGTGCCATTCCTGGACCATCGCTTCTCTTCATACTACCTCTCCTTGCCTCCCGAAATGAGAATCCCTAAGAACGGTATTGAGAAACACCTCTTGCGCGAAACCTTCGAGGACTCCAACCTGATCCCAAAGGAAATTCTCTGGCGTCCGAAAGAGGCTTTCAGCGATGGCATCACTTCCGTGAAGAACAGCTGGTTCAAAATTCTGCAGGAATACGTTGAGCACCAAGTGGACGATGCAATGATGGCGAACGCAGCGCAGAAGTTCCCATTCAACACACCGAAGACGAAAGAAGGCTACTACTACCGTCAAGTCTTCGAGAGACACTACCCTGGACGCGCTGACTGGCTGTCCCATTACTGGATGCCTAAGTGGATCAACGCAACCGATCCCTCTGCGAGGACCCTGACTCACTACAAGTCAGCCGTGAAAGCTGCCGGAGAGAACCTCTACTTCCAGAGCCACCATCATCACCACCATCACCATCACCATTGA

**Human ASNS-TEV-His_10_ (T336I Variant)**

ATGTGTGGTATTTGGGCATTGTTCGGCAGCGACGATTGCTTGTCAGTCCAGTGTCTGTCAGCCATGAAGATTGCTCACCGTGGTCCTGACGCATTCCGTTTCGAAAACGTCAACGGATACACTAACTGCTGTTTCGGTTTCCACAGATTGGCTGTGGTCGACCCTCTGTTCGGTATGCAGCCCATCCGCGTGAAGAAATACCCCTACCTGTGGCTCTGCTACAACGGCGAGATCTACAACCACAAGAAAATGCAGCAACATTTCGAATTCGAGTACCAGACTAAGGTGGACGGAGAAATCATTTTGCACCTGTACGATAAGGGTGGCATCGAGCAAACAATTTGTATGCTGGACGGTGTCTTCGCATTCGTTCTGCTCGATACAGCGAACAAGAAAGTGTTCCTGGGTCGTGACACGTACGGCGTCAGGCCTCTCTTCAAGGCCATGACCGAAGATGGCTTCTTGGCTGTCTGCAGTGAGGCCAAGGGACTGGTTACCCTCAAACACTCGGCTACTCCTTTCCTCAAGGTCGAACCTTTCTTGCCCGGTCATTACGAGGTTCTCGACTTGAAGCCCAACGGCAAAGTCGCATCTGTTGAAATGGTGAAGTACCACCATTGCCGCGACGTTCCCTTGCACGCGCTGTACGATAACGTGGAGAAGCTGTTCCCAGGCTTCGAAATCGAGACGGTTAAGAACAACCTCCGTATTTTGTTCAACAACGCTGTGAAGAAAAGGCTGATGACAGACCGCCGTATCGGATGTTTGCTGAGTGGAGGTCTGGATTCCAGCCTCGTCGCTGCCACGCTCTTGAAGCAGCTCAAAGAAGCTCAGGTTCAATACCCATTGCAAACCTTCGCCATCGGTATGGAGGACTCACCGGATCTGCTCGCAGCGCGCAAGGTGGCTGACCACATCGGTTCCGAACATTACGAGGTCCTGTTCAACAGCGAGGAAGGTATCCAGGCTTTGGACGAAGTCATTTTCTCTCTGGAGACATACGATATCAtCACTGTGCGTGCCTCTGTCGGAATGTACCTCATCTCAAAGTACATTAGGAAAAACACTGACTCAGTTGTGATCTTCAGTGGCGAAGGATCGGATGAGCTGACACAAGGCTACATTTACTTCCACAAGGCTCCAAGTCCGGAAAAAGCCGAGGAAGAGTCGGAAAGGTTGCTGAGAGAGCTGTACCTCTTCGACGTCTTGAGAGCAGATCGTACAACGGCTGCACACGGATTGGAGCTGAGAGTGCCATTCCTGGACCATCGCTTCTCTTCATACTACCTCTCCTTGCCTCCCGAAATGAGAATCCCTAAGAACGGTATTGAGAAACACCTCTTGCGCGAAACCTTCGAGGACTCCAACCTGATCCCAAAGGAAATTCTCTGGCGTCCGAAAGAGGCTTTCAGCGATGGCATCACTTCCGTGAAGAACAGCTGGTTCAAAATTCTGCAGGAATACGTTGAGCACCAAGTGGACGATGCAATGATGGCGAACGCAGCGCAGAAGTTCCCATTCAACACACCGAAGACGAAAGAAGGCTACTACTACCGTCAAGTCTTCGAGAGACACTACCCTGGACGCGCTGACTGGCTGTCCCATTACTGGATGCCTAAGTGGATCAACGCAACCGATCCCTCTGCGAGGACCCTGACTCACTACAAGTCAGCCGTGAAAGCTGCCGGAGAGAACCTCTACTTCCAGAGCCACCATCATCACCACCATCACCATCACCATTGA

**Human ASNS-TEV-His_10_ (F361V Variant)**

ATGTGTGGTATTTGGGCATTGTTCGGCAGCGACGATTGCTTGTCAGTCCAGTGTCTGTCAGCCATGAAGATTGCTCACCGTGGTCCTGACGCATTCCGTTTCGAAAACGTCAACGGATACACTAACTGCTGTTTCGGTTTCCACAGATTGGCTGTGGTCGACCCTCTGTTCGGTATGCAGCCCATCCGCGTGAAGAAATACCCCTACCTGTGGCTCTGCTACAACGGCGAGATCTACAACCACAAGAAAATGCAGCAACATTTCGAATTCGAGTACCAGACTAAGGTGGACGGAGAAATCATTTTGCACCTGTACGATAAGGGTGGCATCGAGCAAACAATTTGTATGCTGGACGGTGTCTTCGCATTCGTTCTGCTCGATACAGCGAACAAGAAAGTGTTCCTGGGTCGTGACACGTACGGCGTCAGGCCTCTCTTCAAGGCCATGACCGAAGATGGCTTCTTGGCTGTCTGCAGTGAGGCCAAGGGACTGGTTACCCTCAAACACTCGGCTACTCCTTTCCTCAAGGTCGAACCTTTCTTGCCCGGTCATTACGAGGTTCTCGACTTGAAGCCCAACGGCAAAGTCGCATCTGTTGAAATGGTGAAGTACCACCATTGCCGCGACGTTCCCTTGCACGCGCTGTACGATAACGTGGAGAAGCTGTTCCCAGGCTTCGAAATCGAGACGGTTAAGAACAACCTCCGTATTTTGTTCAACAACGCTGTGAAGAAAAGGCTGATGACAGACCGCCGTATCGGATGTTTGCTGAGTGGAGGTCTGGATTCCAGCCTCGTCGCTGCCACGCTCTTGAAGCAGCTCAAAGAAGCTCAGGTTCAATACCCATTGCAAACCTTCGCCATCGGTATGGAGGACTCACCGGATCTGCTCGCAGCGCGCAAGGTGGCTGACCACATCGGTTCCGAACATTACGAGGTCCTGTTCAACAGCGAGGAAGGTATCCAGGCTTTGGACGAAGTCATTTTCTCTCTGGAGACATACGATATCACCACTGTGCGTGCCTCTGTCGGAATGTACCTCATCTCAAAGTACATTAGGAAAAACACTGACTCAGTTGTGATCgTCAGTGGCGAAGGATCGGATGAGCTGACACAAGGCTACATTTACTTCCACAAGGCTCCAAGTCCGGAAAAAGCCGAGGAAGAGTCGGAAAGGTTGCTGAGAGAGCTGTACCTCTTCGACGTCTTGAGAGCAGATCGTACAACGGCTGCACACGGATTGGAGCTGAGAGTGCCATTCCTGGACCATCGCTTCTCTTCATACTACCTCTCCTTGCCTCCCGAAATGAGAATCCCTAAGAACGGTATTGAGAAACACCTCTTGCGCGAAACCTTCGAGGACTCCAACCTGATCCCAAAGGAAATTCTCTGGCGTCCGAAAGAGGCTTTCAGCGATGGCATCACTTCCGTGAAGAACAGCTGGTTCAAAATTCTGCAGGAATACGTTGAGCACCAAGTGGACGATGCAATGATGGCGAACGCAGCGCAGAAGTTCCCATTCAACACACCGAAGACGAAAGAAGGCTACTACTACCGTCAAGTCTTCGAGAGACACTACCCTGGACGCGCTGACTGGCTGTCCCATTACTGGATGCCTAAGTGGATCAACGCAACCGATCCCTCTGCGAGGACCCTGACTCACTACAAGTCAGCCGTGAAAGCTGCCGGAGAGAACCTCTACTTCCAGAGCCACCATCATCACCACCATCACCATCACCATTGA

**Human ASNS-TEV-His_10_ (E364A Variant)**

ATGTGTGGTATTTGGGCATTGTTCGGCAGCGACGATTGCTTGTCAGTCCAGTGTCTGTCAGCCATGAAGATTGCTCACCGTGGTCCTGACGCATTCCGTTTCGAAAACGTCAACGGATACACTAACTGCTGTTTCGGTTTCCACAGATTGGCTGTGGTCGACCCTCTGTTCGGTATGCAGCCCATCCGCGTGAAGAAATACCCCTACCTGTGGCTCTGCTACAACGGCGAGATCTACAACCACAAGAAAATGCAGCAACATTTCGAATTCGAGTACCAGACTAAGGTGGACGGAGAAATCATTTTGCACCTGTACGATAAGGGTGGCATCGAGCAAACAATTTGTATGCTGGACGGTGTCTTCGCATTCGTTCTGCTCGATACAGCGAACAAGAAAGTGTTCCTGGGTCGTGACACGTACGGCGTCAGGCCTCTCTTCAAGGCCATGACCGAAGATGGCTTCTTGGCTGTCTGCAGTGAGGCCAAGGGACTGGTTACCCTCAAACACTCGGCTACTCCTTTCCTCAAGGTCGAACCTTTCTTGCCCGGTCATTACGAGGTTCTCGACTTGAAGCCCAACGGCAAAGTCGCATCTGTTGAAATGGTGAAGTACCACCATTGCCGCGACGTTCCCTTGCACGCGCTGTACGATAACGTGGAGAAGCTGTTCCCAGGCTTCGAAATCGAGACGGTTAAGAACAACCTCCGTATTTTGTTCAACAACGCTGTGAAGAAAAGGCTGATGACAGACCGCCGTATCGGATGTTTGCTGAGTGGAGGTCTGGATTCCAGCCTCGTCGCTGCCACGCTCTTGAAGCAGCTCAAAGAAGCTCAGGTTCAATACCCATTGCAAACCTTCGCCATCGGTATGGAGGACTCACCGGATCTGCTCGCAGCGCGCAAGGTGGCTGACCACATCGGTTCCGAACATTACGAGGTCCTGTTCAACAGCGAGGAAGGTATCCAGGCTTTGGACGAAGTCATTTTCTCTCTGGAGACATACGATATCACCACTGTGCGTGCCTCTGTCGGAATGTACCTCATCTCAAAGTACATTAGGAAAAACACTGACTCAGTTGTGATCTTCAGTGGCGcAGGATCGGATGAGCTGACACAAGGCTACATTTACTTCCACAAGGCTCCAAGTCCGGAAAAAGCCGAGGAAGAGTCGGAAAGGTTGCTGAGAGAGCTGTACCTCTTCGACGTCTTGAGAGCAGATCGTACAACGGCTGCACACGGATTGGAGCTGAGAGTGCCATTCCTGGACCATCGCTTCTCTTCATACTACCTCTCCTTGCCTCCCGAAATGAGAATCCCTAAGAACGGTATTGAGAAACACCTCTTGCGCGAAACCTTCGAGGACTCCAACCTGATCCCAAAGGAAATTCTCTGGCGTCCGAAAGAGGCTTTCAGCGATGGCATCACTTCCGTGAAGAACAGCTGGTTCAAAATTCTGCAGGAATACGTTGAGCACCAAGTGGACGATGCAATGATGGCGAACGCAGCGCAGAAGTTCCCATTCAACACACCGAAGACGAAAGAAGGCTACTACTACCGTCAAGTCTTCGAGAGACACTACCCTGGACGCGCTGACTGGCTGTCCCATTACTGGATGCCTAAGTGGATCAACGCAACCGATCCCTCTGCGAGGACCCTGACTCACTACAAGTCAGCCGTGAAAGCTGCCGGAGAGAACCTCTACTTCCAGAGCCACCATCATCACCACCATCACCATCACCATTGA

**Human ASNS-TEV-His_10_ (E364Q Variant)**

ATGTGTGGTATTTGGGCATTGTTCGGCAGCGACGATTGCTTGTCAGTCCAGTGTCTGTCAGCCATGAAGATTGCTCACCGTGGTCCTGACGCATTCCGTTTCGAAAACGTCAACGGATACACTAACTGCTGTTTCGGTTTCCACAGATTGGCTGTGGTCGACCCTCTGTTCGGTATGCAGCCCATCCGCGTGAAGAAATACCCCTACCTGTGGCTCTGCTACAACGGCGAGATCTACAACCACAAGAAAATGCAGCAACATTTCGAATTCGAGTACCAGACTAAGGTGGACGGAGAAATCATTTTGCACCTGTACGATAAGGGTGGCATCGAGCAAACAATTTGTATGCTGGACGGTGTCTTCGCATTCGTTCTGCTCGATACAGCGAACAAGAAAGTGTTCCTGGGTCGTGACACGTACGGCGTCAGGCCTCTCTTCAAGGCCATGACCGAAGATGGCTTCTTGGCTGTCTGCAGTGAGGCCAAGGGACTGGTTACCCTCAAACACTCGGCTACTCCTTTCCTCAAGGTCGAACCTTTCTTGCCCGGTCATTACGAGGTTCTCGACTTGAAGCCCAACGGCAAAGTCGCATCTGTTGAAATGGTGAAGTACCACCATTGCCGCGACGTTCCCTTGCACGCGCTGTACGATAACGTGGAGAAGCTGTTCCCAGGCTTCGAAATCGAGACGGTTAAGAACAACCTCCGTATTTTGTTCAACAACGCTGTGAAGAAAAGGCTGATGACAGACCGCCGTATCGGATGTTTGCTGAGTGGAGGTCTGGATTCCAGCCTCGTCGCTGCCACGCTCTTGAAGCAGCTCAAAGAAGCTCAGGTTCAATACCCATTGCAAACCTTCGCCATCGGTATGGAGGACTCACCGGATCTGCTCGCAGCGCGCAAGGTGGCTGACCACATCGGTTCCGAACATTACGAGGTCCTGTTCAACAGCGAGGAAGGTATCCAGGCTTTGGACGAAGTCATTTTCTCTCTGGAGACATACGATATCACCACTGTGCGTGCCTCTGTCGGAATGTACCTCATCTCAAAGTACATTAGGAAAAACACTGACTCAGTTGTGATCTTCAGTGGCcAAGGATCGGATGAGCTGACACAAGGCTACATTTACTTCCACAAGGCTCCAAGTCCGGAAAAAGCCGAGGAAGAGTCGGAAAGGTTGCTGAGAGAGCTGTACCTCTTCGACGTCTTGAGAGCAGATCGTACAACGGCTGCACACGGATTGGAGCTGAGAGTGCCATTCCTGGACCATCGCTTCTCTTCATACTACCTCTCCTTGCCTCCCGAAATGAGAATCCCTAAGAACGGTATTGAGAAACACCTCTTGCGCGAAACCTTCGAGGACTCCAACCTGATCCCAAAGGAAATTCTCTGGCGTCCGAAAGAGGCTTTCAGCGATGGCATCACTTCCGTGAAGAACAGCTGGTTCAAAATTCTGCAGGAATACGTTGAGCACCAAGTGGACGATGCAATGATGGCGAACGCAGCGCAGAAGTTCCCATTCAACACACCGAAGACGAAAGAAGGCTACTACTACCGTCAAGTCTTCGAGAGACACTACCCTGGACGCGCTGACTGGCTGTCCCATTACTGGATGCCTAAGTGGATCAACGCAACCGATCCCTCTGCGAGGACCCTGACTCACTACAAGTCAGCCGTGAAAGCTGCCGGAGAGAACCTCTACTTCCAGAGCCACCATCATCACCACCATCACCATCACCATTGA

**Human ASNS-TEV-His_10_ (D367A Variant)**

ATGTGTGGTATTTGGGCATTGTTCGGCAGCGACGATTGCTTGTCAGTCCAGTGTCTGTCAGCCATGAAGATTGCTCACCGTGGTCCTGACGCATTCCGTTTCGAAAACGTCAACGGATACACTAACTGCTGTTTCGGTTTCCACAGATTGGCTGTGGTCGACCCTCTGTTCGGTATGCAGCCCATCCGCGTGAAGAAATACCCCTACCTGTGGCTCTGCTACAACGGCGAGATCTACAACCACAAGAAAATGCAGCAACATTTCGAATTCGAGTACCAGACTAAGGTGGACGGAGAAATCATTTTGCACCTGTACGATAAGGGTGGCATCGAGCAAACAATTTGTATGCTGGACGGTGTCTTCGCATTCGTTCTGCTCGATACAGCGAACAAGAAAGTGTTCCTGGGTCGTGACACGTACGGCGTCAGGCCTCTCTTCAAGGCCATGACCGAAGATGGCTTCTTGGCTGTCTGCAGTGAGGCCAAGGGACTGGTTACCCTCAAACACTCGGCTACTCCTTTCCTCAAGGTCGAACCTTTCTTGCCCGGTCATTACGAGGTTCTCGACTTGAAGCCCAACGGCAAAGTCGCATCTGTTGAAATGGTGAAGTACCACCATTGCCGCGACGTTCCCTTGCACGCGCTGTACGATAACGTGGAGAAGCTGTTCCCAGGCTTCGAAATCGAGACGGTTAAGAACAACCTCCGTATTTTGTTCAACAACGCTGTGAAGAAAAGGCTGATGACAGACCGCCGTATCGGATGTTTGCTGAGTGGAGGTCTGGATTCCAGCCTCGTCGCTGCCACGCTCTTGAAGCAGCTCAAAGAAGCTCAGGTTCAATACCCATTGCAAACCTTCGCCATCGGTATGGAGGACTCACCGGATCTGCTCGCAGCGCGCAAGGTGGCTGACCACATCGGTTCCGAACATTACGAGGTCCTGTTCAACAGCGAGGAAGGTATCCAGGCTTTGGACGAAGTCATTTTCTCTCTGGAGACATACGATATCACCACTGTGCGTGCCTCTGTCGGAATGTACCTCATCTCAAAGTACATTAGGAAAAACACTGACTCAGTTGTGATCTTCAGTGGCGAAGGATCGGcTGAGCTGACACAAGGCTACATTTACTTCCACAAGGCTCCAAGTCCGGAAAAAGCCGAGGAAGAGTCGGAAAGGTTGCTGAGAGAGCTGTACCTCTTCGACGTCTTGAGAGCAGATCGTACAACGGCTGCACACGGATTGGAGCTGAGAGTGCCATTCCTGGACCATCGCTTCTCTTCATACTACCTCTCCTTGCCTCCCGAAATGAGAATCCCTAAGAACGGTATTGAGAAACACCTCTTGCGCGAAACCTTCGAGGACTCCAACCTGATCCCAAAGGAAATTCTCTGGCGTCCGAAAGAGGCTTTCAGCGATGGCATCACTTCCGTGAAGAACAGCTGGTTCAAAATTCTGCAGGAATACGTTGAGCACCAAGTGGACGATGCAATGATGGCGAACGCAGCGCAGAAGTTCCCATTCAACACACCGAAGACGAAAGAAGGCTACTACTACCGTCAAGTCTTCGAGAGACACTACCCTGGACGCGCTGACTGGCTGTCCCATTACTGGATGCCTAAGTGGATCAACGCAACCGATCCCTCTGCGAGGACCCTGACTCACTACAAGTCAGCCGTGAAAGCTGCCGGAGAGAACCTCTACTTCCAGAGCCACCATCATCACCACCATCACCATCACCATTGA

**Human ASNS-TEV-His_10_ (D367N Variant)**

ATGTGTGGTATTTGGGCATTGTTCGGCAGCGACGATTGCTTGTCAGTCCAGTGTCTGTCAGCCATGAAGATTGCTCACCGTGGTCCTGACGCATTCCGTTTCGAAAACGTCAACGGATACACTAACTGCTGTTTCGGTTTCCACAGATTGGCTGTGGTCGACCCTCTGTTCGGTATGCAGCCCATCCGCGTGAAGAAATACCCCTACCTGTGGCTCTGCTACAACGGCGAGATCTACAACCACAAGAAAATGCAGCAACATTTCGAATTCGAGTACCAGACTAAGGTGGACGGAGAAATCATTTTGCACCTGTACGATAAGGGTGGCATCGAGCAAACAATTTGTATGCTGGACGGTGTCTTCGCATTCGTTCTGCTCGATACAGCGAACAAGAAAGTGTTCCTGGGTCGTGACACGTACGGCGTCAGGCCTCTCTTCAAGGCCATGACCGAAGATGGCTTCTTGGCTGTCTGCAGTGAGGCCAAGGGACTGGTTACCCTCAAACACTCGGCTACTCCTTTCCTCAAGGTCGAACCTTTCTTGCCCGGTCATTACGAGGTTCTCGACTTGAAGCCCAACGGCAAAGTCGCATCTGTTGAAATGGTGAAGTACCACCATTGCCGCGACGTTCCCTTGCACGCGCTGTACGATAACGTGGAGAAGCTGTTCCCAGGCTTCGAAATCGAGACGGTTAAGAACAACCTCCGTATTTTGTTCAACAACGCTGTGAAGAAAAGGCTGATGACAGACCGCCGTATCGGATGTTTGCTGAGTGGAGGTCTGGATTCCAGCCTCGTCGCTGCCACGCTCTTGAAGCAGCTCAAAGAAGCTCAGGTTCAATACCCATTGCAAACCTTCGCCATCGGTATGGAGGACTCACCGGATCTGCTCGCAGCGCGCAAGGTGGCTGACCACATCGGTTCCGAACATTACGAGGTCCTGTTCAACAGCGAGGAAGGTATCCAGGCTTTGGACGAAGTCATTTTCTCTCTGGAGACATACGATATCACCACTGTGCGTGCCTCTGTCGGAATGTACCTCATCTCAAAGTACATTAGGAAAAACACTGACTCAGTTGTGATCTTCAGTGGCGAAGGATCGaATGAGCTGACACAAGGCTACATTTACTTCCACAAGGCTCCAAGTCCGGAAAAAGCCGAGGAAGAGTCGGAAAGGTTGCTGAGAGAGCTGTACCTCTTCGACGTCTTGAGAGCAGATCGTACAACGGCTGCACACGGATTGGAGCTGAGAGTGCCATTCCTGGACCATCGCTTCTCTTCATACTACCTCTCCTTGCCTCCCGAAATGAGAATCCCTAAGAACGGTATTGAGAAACACCTCTTGCGCGAAACCTTCGAGGACTCCAACCTGATCCCAAAGGAAATTCTCTGGCGTCCGAAAGAGGCTTTCAGCGATGGCATCACTTCCGTGAAGAACAGCTGGTTCAAAATTCTGCAGGAATACGTTGAGCACCAAGTGGACGATGCAATGATGGCGAACGCAGCGCAGAAGTTCCCATTCAACACACCGAAGACGAAAGAAGGCTACTACTACCGTCAAGTCTTCGAGAGACACTACCCTGGACGCGCTGACTGGCTGTCCCATTACTGGATGCCTAAGTGGATCAACGCAACCGATCCCTCTGCGAGGACCCTGACTCACTACAAGTCAGCCGTGAAAGCTGCCGGAGAGAACCTCTACTTCCAGAGCCACCATCATCACCACCATCACCATCACCATTGA

**Human His_10_-3C-ASS1**

ATGCATCACCATCATCACCACCATCACCACCATCTGGAAGTTCTGTTCCAGGGGCCCggatccATGTCCAGCAAGGGTTCCGTGGTCCTGGCTTACAGCGGTGGCCTGGACACCTCTTGCATCCTGGTGTGGCTGAAGGAGCAGGGCTACGACGTCATCGCTTACCTGGCCAACATCGGACAGAAGGAAGACTTCGAGGAAGCTCGTAAGAAGGCCCTGAAGCTGGGCGCCAAGAAGGTGTTCATCGAGGACGTCTCTAGGGAATTCGTGGAGGAATTCATCTGGCCAGCTATCCAGTCTTCAGCCCTGTACGAGGACAGGTACCTGCTGGGAACCAGCCTGGCTCGCCCTTGCATCGCCCGTAAGCAGGTGGAGATCGCTCAGAGAGAAGGTGCCAAGTACGTCTCTCACGGCGCTACTGGAAAGGGTAACGACCAGGTGCGCTTCGAACTGTCCTGCTACAGCCTGGCTCCTCAGATCAAGGTCATCGCCCCATGGCGTATGCCTGAGTTCTACAACAGGTTCAAGGGAAGAAACGACCTGATGGAATACGCTAAGCAGCACGGTATCCCAATCCCTGTGACCCCCAAGAACCCATGGTCAATGGACGAGAACCTGATGCACATCTCCTACGAGGCTGGCATCCTGGAAAACCCCAAGAACCAGGCCCCTCCCGGACTGTACACCAAGACTCAGGACCCAGCTAAGGCCCCTAACACTCCCGACATCCTGGAGATCGAATTCAAGAAGGGTGTGCCCGTCAAGGTGACCAACGTGAAGGACGGCACCACTCACCAGACTTCACTGGAGCTGTTCATGTACCTGAACGAAGTCGCTGGAAAGCACGGCGTGGGACGCATCGACATCGTCGAGAACCGTTTCATCGGAATGAAGTCCAGGGGTATCTACGAAACCCCCGCCGGTACTATCCTGTACCACGCTCACCTGGACATCGAGGCCTTCACCATGGACCGCGAAGTGCGTAAGATCAAGCAGGGTCTGGGCCTGAAGTTCGCTGAGCTGGTCTACACTGGTTTCTGGCACAGCCCAGAGTGCGAATTCGTGAGACACTGCATCGCCAAGAGCCAGGAGCGCGTCGAAGGCAAGGTCCAGGTGTCTGTCCTGAAGGGCCAGGTGTACATCCTGGGACGTGAGTCTCCTCTGTCACTGTACAACGAGGAACTGGTGTCCATGAACGTCCAGGGAGACTACGAGCCCACCGACGCTACTGGTTTCATCAACATCAACTCACTGAGGCTGAAGGAATACCACAGACTGCAGTCCAAGGTCACTGCCAAGTAA
